# Effects of plant-based medicinal food on postoperative recurrence and lung metastasis of gastric cancer regulated by Wnt/β-catenin-EMT signaling pathway and VEGF-C/D-VEGFR-3 cascade in a mouse model

**DOI:** 10.1186/s12906-022-03703-0

**Published:** 2022-09-02

**Authors:** Lin Tian, Xuxi Chen, Li Cao, Lishi Zhang, Jinyao Chen

**Affiliations:** 1 West China School of Public Health and West China Fourth Hospital, Sichuan University, Chengdu, China; 2 Food Safety Monitoring and Risk Assessment Key Laboratory of Sichuan Province, Chengdu, China

**Keywords:** Gastric cancer, Medicinal food, Distant metastasis, Epithelial–mesenchymal transition, Wnt/β–catenin signaling pathway, VEGF-C/D-VEGFR-3 signaling cascade

## Abstract

**Background:**

The plant-based medicinal food (PBMF) is a functional compound extracted from 6 medicinal and edible plants: Coix seed, *L. edodes*, *A. officinalis* L., *H. cordata*, *Dandelion*, and *G. frondosa*. Our previous studies have confirmed that the PBMF possesses anti-tumor properties in a subcutaneous xenograft model of nude mice. This study aims to further investigate the effects and potential molecular mechanisms of the PBMF on the recurrence and metastasis of gastric cancer (GC).

**Methods:**

Postoperative recurrence and metastasis model of GC was successfully established in inbred 615 mice inoculated with mouse forestomach carcinoma (MFC) cells. After tumorectomy, 63 GC mice were randomly divided into five groups and respectively subject to different treatments for 15 days as below: model control group, 5-Fu group, and three doses of PBMF (43.22, 86.44, 172.88 g/kg PBMF in diet respectively). The inhibition rate (IR) of recurrence tumor weights and organ coefficients were calculated. Meanwhile, histopathological changes were examined and the metastasis IR in lungs and lymph node tissues was computed. The mRNA expressions related to the canonical Wnt/β-catenin signaling pathway, epithelial-mesenchymal transition (EMT) and lymphangiogenesis were detected by RT-qPCR in recurrence tumors and/or lung tissues. Protein expressions of β-catenin, p-β-catenin (Ser33/37/Thr41), GSK-3β, p-GSK-3β (Ser9), E-cadherin, and Vimentin in recurrence tumors were determined by Western Blot. LYVE-1, VEGF-C/D, and VEGFR-3 levels in recurrence tumors and/or lung tissues were determined by immunohistochemistry staining.

**Results:**

The mRNA, as well as protein expression of GSK-3β were up-regulated and the mRNA expression of β-catenin was down-regulated after PBMF treatment. Meanwhile, the ratio of p-β-catenin (Ser33/37/Thr41) to β-catenin protein was increased significantly and the p-GSK-3β (Ser9) protein level was decreased. And PMBF could effectively decrease the mRNA and protein levels of Vimentin while increasing those of E-cadherin. Furthermore, PBMF markedly reduced lymphatic vessel density (LVD) (labeled by LYVE-1) in recurrence tumor tissues, and mRNA levels of VEGF-C/D, VEGFR-2/3 of recurrence tumors were all significantly lower in the high-dose group.

**Conclusions:**

PBMF had a significant inhibitory effect on recurrence and lung metastasis of GC. The potential mechanism may involve reversing EMT by inhabiting the Wnt/β-catenin signaling pathway. Lymphatic metastasis was also inhibited by PBMF via down-regulating the activation of the VEGF-C/D-VEGFR-2/3 signaling cascade.

**Supplementary Information:**

The online version contains supplementary material available at 10.1186/s12906-022-03703-0.

## Introduction

Gastric cancer (GC) is the fifth most prevalent malignancy worldwide. Epidemiological data from the International Agency for Research on Cancer (IARC) indicated that the estimated age-standardized incidence rate of GC was 11.1% with 1,089,103 new cases, and GC was the fourth leading cause of cancer-related mortality in 2020 globally. In China, GC is more prevalent and with higher mortality. In 2020, about 478,508 new cases were diagnosed, and 373,789 people died of GC, accounting for about 44% of the global morbidity and 49% of GC mortality. At present, surgical resection of the tumor is the main treatment for GC, but distant metastasis and postoperative recurrence still occur quite frequently. Approximately 50% of GC patients suffered from recurrence after surgical resection, even more than 90% of cancer deaths were not due to primary tumors but to metastases [1, 2]. Moreover, the five-year survival rate of patients with advanced gastric cancer (AGC) is only about 30% in a specific geographical region in China [3]. There is an urgent need to find effective therapeutic strategies to improve the prognosis and life quality of patients with GC.

Studies indicated that natural products such as phytochemicals and Traditional Chinese Medicine (TCM) had significant anti-tumor potential. About 50% of approved anti-tumor drugs from 1940 to 2014 originate from natural products or derive therefrom, including paclitaxel, vincristine, irinotecan, etoposide, and other first-line chemotherapy drugs [4].

Modern pharmacological studies have found that the *Coix* seed can achieve direct anti-tumor and immune regulation through multi-target and multi-channel synergy, such as inhibiting tumor growth by down-regulating the expression of vascular endothelial growth factor (VEGF) and basic fibroblast growth factor (bFGF) protein to block the angiogenesis of S180 sarcoma, and its inhibition rate (IR) reached 46.21% [5]. Polysaccharide from *L. edodes* has been successfully used as an adjuvant chemotherapy drug to treat lymphatic metastasis in malignancies, such as colorectal cancer, lung cancer, and gastric cancer [6]. Meanwhile, Latcripin 1 (LP1) from the *L. edodes* strain C_91-3_ slows the pace of migration and invasion in SGC-7901 cells by suppressing the expression level of MMP-2 and MMP-9 [7]. Lentinula edodes mycelia extract (LEM) can relieve adverse effects from chemotherapy in AGC patients [8]. *A. officinalis* L. and its extracts containing abundant asparagine, aspartic acid, and other saponins have obvious anti-tumor activity, which also can prevent the invasion of cancer cells [9, 10]. *H. cordata*, *Dandelion*, and *G. frondosa* all possess the characteristic against cancer [11–17]. Multi-targeting agents present additive or synergistic effects and can address these challenges of limited efficiency, poor safety, and drug resistance of single target agents [18]. Hence, the combination of these plants can plausibly be a more effective strategy against GC.

Anti-tumor effects of the above substances have been supported, but their combined effects and potential mechanisms are still unclear and need further illuminated. EMT is a critical event in the invasion and metastasis of GC. Epithelial-derived gastric cancer cells lose intercellular adhesion and polarity, and in turn transform from stable epithelial cells to mesenchymal cells with strong activity, finally enhancing their migration and invasion ability, which are the typical course of EMT [19]. Wnt/β-catenin signaling pathway plays an important role in the occurrence, development, postoperative recurrence, invasion, and metastasis of GC [20]. It has been reported that dysregulation of the Wnt/β-catenin signaling pathway can be observed in nearly 40% of GC patients, suggesting that it possibly be a potential target for the prevention and treatment of GC [21]. Wnt/β-catenin and EMT pathways are interactional. The down-regulation of E-cadherin expressed on epithelial cell surfaces in the EMT process would activate the Wnt/β-catenin signaling pathway. The inhibition of Wnt/β-catenin signaling can also prevent the occurrence of EMT and thus restrain tumor metastasis [22].

Lymphatic metastasis is one of the most important criteria to evaluate the prognosis of GC patients. The Union for International Cancer Control (UICC) also proposed that lymphangiogenesis rate and lymph node metastasis number can be used as key indicators to predict the prognosis of GC [23, 24]. Lymphatic vessel neogenesis around tumor cells is the basis of lymphatic metastasis, which is mainly regulated by two members of the VEGF family (VEGF-C and VEGF-D) and their receptor VEGFR-2/3 [25]. In addition, LYVE-1 is a lymphatic endothelial hyaluronic acid receptor, as well as a homologous compound of CD44 glycoprotein. It is expressed in lymphatic endothelial cells and is regarded as a relatively specific marker of lymphatic endothelial cells [26].

Our previous studies have reported that the same plant-based medicinal food (PBMF) consisting of the above medicinal and edible plants can prominently suppress the growth of subcutaneous xenograft tumors in nude mice with fewer side effects [27]. Nevertheless, distant metastasis rarely occurs in the subcutaneous xenograft model. Accordingly, in this study, we established the postoperative recurrence and metastasis model in 615 mice using mouse forestomach carcinoma (MFC) cells, and the beneficial effects against GC of PBMF were further investigated.

## Materials and methods

The PBMF was prepared by Jiangxi Gongqing Jiangzhong Dietary Therapy Technology Ltd (Jiujiang China). It was a mixture of 6 plant extracts, which consist of 12 g *Coix* seed, 12 g *L. edodes*, 12 g *A. officinalis* L., 12 g *H. cordata*, 12 g *Dandelion*, and 9 g *G. frondosa* [22]. Table 1 shows the full scientific species (Latin binomial nomenclature) names of all ingredients. The provided names can be verified in the Chinese Field Herbarium (www.cfh.ac.cn/).

**Table 1:** The full scientific species names of all plants in PBMF

Species of plants	Latin binomial nomenclature
*Coix* seed	*Coix lacryma-jobi* Linn
*L. edodes*	*Lentinus edodes* (Berk.) Pegler 1976
*A. officinalis* L	*Asparagus officinalis* Linn
*H. cordata*	*Houttuynia cordata* Thunb
*Dandelion*	*Taraxacum mongolicum* Hand.-Mazz., 1907
*G. frondosa*	*Grifola frondosa* (Dicks.) Gray 1821

### Cell culture

MFC cells were obtained from KeyGEN Biotechnology (Jiangsu China). Cells were cultured in DMEM (High Glucose) (Hyclone, USA) supplemented with 10% (v/v) fetal bovine serum (Gibco, USA) and 1% (v/v) penicillin/streptomycin (Hyclone, USA) and incubated in 5% CO_2_ at 37 °C atmosphere.

### Animal care

Sixty-five Specific Pathogen Free (SPF) male mice (strain 615) aged 4–5 weeks with body weights of 16–18 g were purchased from Laboratory Animal Center, Institute of Hematology, Chinese Academy of Medical Sciences (Tianjin China) (Permit No.: SCXK [jin] 2015–0001). Mice were raised under the SPF environment (23–24 °C, 50–60% humidity) with a 12/12 h light/dark cycle in the Analysis and Testing Center of West China School of Public Health (Permit No.: SYXK [chuan] 2018–011). Animal experiments were carried out strictly by the Guide for the Care and Use of Laboratory Animals of Sichuan University and approved by the Ethics Committee of West China Fourth Hospital and West China School of Public Health (Animal ethical approval No.: Gwll2021049). All mice were fed with basic pellet diet for 1 week before the experiments.

### Establishment of postoperative recurrence and metastasis model

MFC cells were resuspended with serum- and antibiotic-free DMEM to adjust cell concentration to 4 × 10^7^ cells/mL. Cells (50 μL) were subcutaneously injected into the footpad of the left hind leg. The tumor growth was monitored every day. After 10 days, phymas (primary tumors) were palpable at the inoculation site and the degree of the invasion reached grade III-IV (Table 2). At this time, the left limbs of the tumor-bearing mice were surgically amputated with the 3 mm above tumors to remove primary tumors. There need to be some residual tumor cells on the cut surface after sterile surgery, mimicking the status of AGC patients undergoing surgical resection.

**Table 2:** Invasion grading of subcutaneous xenograft tumor in the footpad [28]

Grading	Invasion representation
Grade 0	Tumor cells begin to proliferate at the inoculation site. The tumor margin is neat and tumor cells do not invade surrounding tissues
Grade I	There is mild edema around the tumor tissue and a small number of inflammatory cells infiltrated. A single tumor cell begins to invade surrounding tissues and host tissues do not change
Grade II	The growth of tumor cells is active and surrounding tissues have evident edema. A large number of inflammatory cells surround the tumor body. Groups of tumor cells invade muscle tissues, but degeneration and atrophy are not observed
Grade III	Tumor cells invade muscle tissues diffusely and muscle cells appear degeneration and atrophy. The edema and inflammation around the tumor are more evident. The center of the tumor appears necrosis. Meanwhile, tumor cells have invaded the dermis, but atrophy or thinning is not observed in the epidermis
Grade IV	Tumor cells invade subcutaneous tissues diffusely and some invade the epidermis, making the epidermis thinner or broken. Most of the muscle tissues and some bone tissues are invaded, and there is extensive necrosis in the center of the tumor

### Treatment of model mice

The general status and survival of all the model mice were observed the next day after the resection of the primary tumor. Except for two mice that died from their intolerance to the operation, other mice were in good condition without any abnormality. Then sixty-three well-established model mice were randomly divided into five groups with eleven mice in the 5-Fu group and thirteen mice in other groups: Model control group (normal diet), 5-Fu group (normal diet), low-dose group (a particular diet containing the PBMF at a concentration of 43.22 g/kg diet), medium-dose group (86.44 g/kg PBMF diet), and a high-dose group (172.88 g/kg PBMF diet). 5-Fu group was induced by intraperitoneal injection of 0.2 mL 5-Fu (Sigma Aldrich, USA) [20 mg/kg] and other groups were replaced by 0.2 mL saline.

Diet intervention started on the day of random grouping and lasted for fifteen days. Saline and 5-Fu were intraperitoneally administered every day for the first week (7 days). Body weights were recorded twice every week. All mice were euthanized by cervical dislocation after fasting overnight at the end of the experiments. Heart, liver, spleen, lung, and kidney were weighted and the organ coefficient was calculated by the formula:

$$\mathrm{organ\;coefficient}=(\mathrm{organ\;wet\;weight}/\mathrm{body\;weight})\times100\%$$

Recurrence tumors were harvested and weighed. IR was computed using the formula:

$$\mathrm{IR}\ \mathrm{of}\ \mathrm{recurrence}\ \mathrm{tumor}\ \mathrm{weight}=\left(1-{\mathrm{W}}_{\mathrm{T}}/{\mathrm{W}}_{\mathrm{C}}\right)\times 100\%,$$

where W_T_ is the mean recurrence tumor weight of treatment groups, and W_C_ is the mean recurrence tumor weight of the model control group.

Portions of recurrence tumor tissues were fixed in 4% paraformaldehyde solution for 24 h, which were embedded in paraffin, sectioned, and then subjected to hematoxylin and eosin (H&E) staining and immunohistochemistry analysis. Another portion was immediately stored at − 80 °C for subsequent RNA extraction and Western Blot analysis. Heart, liver, spleen, kidney, and lymph node tissues were fixed in 4% paraformaldehyde solution lasted 24 h for H&E staining. One lobe of lung tissue was cut and stored at − 80 °C for subsequent RNA extraction. The remaining lung tissue was fixed in 4% paraformaldehyde solution and lasted 24 h for H&E staining and immunohistochemistry analysis.

### Histopathological examination

#### Histopathological examination of recurrence tumor tissues

Paraffin-embedded blocks of recurrence tissues were cut into 4 μm-thick sections and followed routine H&E staining. The histomorphology of tumor cells was observed at 200 × magnification under the optical microscope (Olympus, Tokyo, Japan, BX53).

#### Observation of lung metastases and calculation of metastasis IR

On the day of dissection, bilateral lung tissues were observed to whether there were obvious metastases on the surface. Subsequently, the lung tissue was fixed, embedded, and sectioned for H&E staining. Lung metastases were observed under the microscope and graded according to the size and number of metastases, as shown in Table 3. The total score and IR of pulmonary metastasis were calculated as follows:

**Table 3:** Histological grading of lung metastasis based on nodular metastasis [28]

Grading	Pathological features	score
Grade 0	Without metastasis	0
Grade I	There are 1 ~ 5 small metastases less than 1 mm in diameters in the lung and a mitotic figure is occasionally seen in the metastasis	1
Grade II	There are 5 ~ 10 small metastases or 1 ~ 2 intermediate metastases between 1 ~ 2 mm in diameter	2
Grade III	More than 10 small metastases are widely distributed or large metastases more than 2 mm in diameter occur in the lung	3

$$\mathrm{Total\;pulmonary\;metastasis\;score }=\mathrm{\;number\;of\;grade\;I }\times\;1\;+\mathrm{\;number\;of\;grade\;II }\times\;2\;+\mathrm{\;number\;of\;grade\;III }\times\;3$$

$$\mathrm{IR}\ \mathrm{of}\ \mathrm{pulmonary}\ \mathrm{metastasis}=\left(1-{\mathrm{PS}}_{\mathrm{T}}/{\mathrm{PS}}_{\mathrm{C}}\right)\times 100\%,$$

where PS_T_ is the mean pulmonary metastasis score of treatment groups, and PS_C_ is the mean pulmonary metastasis score of the model control group.

#### Observation of lymph node metastases and calculation of metastasis inhibition rate

Bilateral popliteal, inguinal, and axillary lymph nodes metastases were observed by routine H&E staining. Histological grade refers to Table 4. The total score and IR of lymph node metastasis were calculated as follows:

**Table 4:** Histological grading of lymph node metastases [28]

Grading	Pathological features	score
Grade 0	Without metastasis	0
Grade I	There are piles of tumor cells only in the marginal sinus of lymph nodes, forming small metastases, and sometimes mitotic figure is seen	1
Grade II	There are obvious metastases both in the marginal and central sinus of lymph nodes	2
Grade III	Lymph nodes are mostly filled with tumor cells and common lesions can be observed in metastases	3
Grade IV	More than one lymph node has metastases, forming extensive metastases	4

$$\mathrm{Total}\;\mathrm{lymph}\;\mathrm{node}\;\mathrm{metastasis}\;\mathrm{score}\;=\;\mathrm{number}\;\mathrm{of}\;\mathrm{grade}\;\mathrm I\;\times1\;+\;\mathrm{number}\;\mathrm{of}\;\mathrm{grade}\;\mathrm{II}\;\times\;2\;+\;\mathrm{number}\;\mathrm{of}\;\mathrm{grade}\;\mathrm{III}\;\times\;3\;+\;\mathrm{number}\;\mathrm{of}\;\mathrm{grade}\;\mathrm{IV}\;\times\;4$$

$$\mathrm{IR}\ \mathrm{of}\ \mathrm{lymph}\ \mathrm{node}\ \mathrm{metastasis}=\left(1-{\mathrm{LS}}_{\mathrm{T}}/{\mathrm{LS}}_{\mathrm{C}}\right)\times 100\%,$$

where LS_T_ is the mean lymph node metastasis score of treatment groups, and LS_C_ is the mean lymph node metastasis score of the model control group

### RT-qPCR

Gene expressions of the Wnt/β-catenin signaling pathway, EMT, and lymphangiogenesis-related genes in recurrence tumors and/or lung tissues were detected by RT-qPCR. Total RNA was extracted by Animal Total RNA Isolation Kit (Foregene, China) following the manufacturer’s instructions. The RNA concentration was measured by NanoDrop TM 2000 (Thermo, MA, USA). All samples were adjusted to the same level of 500 ng/μL and reverse-transcribed to cDNA using Iscript cDNA Synthesis Kit (Bio-Rad, USA). The sequences of specific gene primers were synthesized by Sangong Biotech Company (Table 5). GAPDH was used as an internal control. Target genes were amplified using cDNA (1 μL), SsoFast EvaGreen Supermix (5 μL, Bio-Rad, USA), forward primer (0.3 μL), reverse primer (0.3 μL), and RNase-free H_2_O up to 10 μL in a CFX96 Real-Time PCR Detection System (Bio-Rad, USA). Relative mRNA expression levels were analyzed by the 2^−ΔΔCq^ method and calculated as follows [27]:

$$\mathrm{\Delta Cq }=\mathrm{ Cq }\;(\mathrm{target\;gene}) -\mathrm{ Cq }\;(\mathrm{reference\;gene})$$

$$\mathrm{\Delta \Delta Cq }=\mathrm{ \Delta Cq }\;(\mathrm{treatment\;group}) -\mathrm{ \Delta Cq }\;(\mathrm{model\;control\;group})$$

**Table 5:** Primer sequences designed for RT-PCR (5′-3′)

Gene	Sequences (5'-3')
β-catenin	Forward: GGTGGACCCCAAGCCTTAGTA
	Reverse: AGATGAAGCCCCAGTGCCT
GSK-3β	Forward: CCACCATCCTTATCCCTCCA
	Reverse: AGCGGCGTTATTGGTCTGTC
E-cadherin	Forward: GGCTGGACCGAGAGAGTTACC
	Reverse: TGACCTCATTCTCAGGCACTTG
Vimentin	Forward: CCTCTATTCCTCATCCCCCG
	Reverse: AACTCAGTGTTGATGGCGTCG
VEGF-C	Forward: TGTGGGGAAGGAGTTTGGAG
	Reverse: CGGCAGGAAGTGTGATTGG
VEGF-D	Forward: TGCCCGAGTTAGTGCCTGTTA
	Reverse: CTTCTTCTGGGGTCTGAATGGA
VEGFR-2	Forward: GATTTTCTCCATCCCCCCA
	Reverse: GATACTGTCACCACCGCCG
VEGFR-3	Forward: GCCAAGGGCTGCGTAAACT
	Reverse: CCCAGAAGAAAACTGCGATGA
GAPDH	Forward: CCTTCCGTGTTCCTACCCC
	Reverse: GCCCAAGATGCCCTTCAGT

### Western blot

Expression levels of the target protein in recurrence tumors were detected by Western Blot. The samples derived from the same experiment and blots were processed in parallel. Recurrence tumor tissues were removed from − 80 °C and 50 mg of tissue from each sample was ground quickly with liquid nitrogen in the grinding bowl. Total protein was obtained after lying on the powdery tissues with 500 μL cold RIPA (Beyotime Biotechnology, China) containing protease and phosphatase inhibitors (APE × BIO, USA). Protein concentrations were quantified by BCA Protein Assay Kit (KeyGEN Biotechnology, Jiangsu, China). Dilute the protein concentration of samples to the same level with an appropriate amount of RIPA. Then add an appropriate volume of 5 × loading buffer according to the proportion of 4:1 (sample: 5 × loading buffer). After vortex mixing, boil at 100 °C for 5 min for protein denaturation. The whole process needs to be conducted on ice to avoid protein degradation.

The protein was separated by 10% polyacrylamide gel (Baihe Technology, Chengdu, China) and then transferred onto a PVDF transfer membrane (0.45 μm, Millipore, USA). Then, the protein on the membrane was washed with TBS containing 0.1% Tween-20 (TBST) and blocked by 5% skim milk (wt/vol). Considering the cross-reaction, the blots were cut prior to hybridization with antibodies. After washing with PBST, the membrane was incubated with primary antibodies [β-catenin, p-β-catenin(Ser33/37/Thr41), GSK-3β, p-GSK-3β(Ser9), E-cadherin and Vimentin, Cell Signaling Technology] (GAPDH, Proteintech) overnight at 4 °C. The specific phosphorylation sites of p-β-catenin are Ser33/37/Thr41 and p-GSK-3β was Ser9. After being washed, incubating with horseradish peroxidase (HRP)-conjugated secondary antibodies (1: 2000 dilution, Proteintech) for 1 h. Finally, the protein band dipped in highly sensitive chemiluminescence liquid (Millipore, USA) was visualized using Chemiluminescence Imaging System (Bio-Rad, USA). The gray value was analyzed by Image J, and relative expression levels of target proteins were calculated as follows:

$$\mathrm{Relative\;expression }=\mathrm{ gray\;value\;of\;target\;protein }/\mathrm{ gray\;value\;of\;GAPDH}.$$

### Immunohistochemistry staining

Expression levers of LYVE-1, VEGF-C/D, and VEGFR-3 proteins in recurrence tumors and lung tissues were determined by immunohistochemistry. The selected sections were routinely deparaffinized and hydrated. Antigens were retrieved by heating the sections in a buffer (EDTA, pH 9.0; sodium citrate, pH 6.0). Then the section was incubated in 3% H_2_O_2_ and blocked with 5% BSA. Then incubating overnight with primary antibodies specific for LYVE-1 (Servicebio), VEGF-C/D (Proteintech), and Vascular endothelial growth factor receptor-3 (VEGFR-3) (Bioss) and the next day with HRP-conjugated secondary antibodies (Proteintech). Coloration was performed with 3′-diaminobenzidine (DAB) (Servicebio), and the sections were counterstained with hematoxylin (Servicebio). Images were viewed under a microscope (CIC, XSP-C204).

Lymphatic vessel density (LVD) was labeled by LYVE-1, which was stained in a pale brown granule. According to reference [29], the strip, slit-like and tubular cell clusters composed of positive stained single lymphatic endothelial cells or clustered endothelial cells are recorded as positive lymphatic vessels. Furthermore, VEGF-C-, VEGF-D- and VEGFR-3-positive staining were evident in the cytomembrane and cytoplasm, and pale brown tumor cells were regarded as positive cells. Image-pro Plus 6.0 software was used to calculate the average integrated optical density (IOD) per stained area (IOD/area) for positive staining.

### Statistical analysis

Data were analyzed using SPSS 21.0 and expressed as mean ± standard deviation (SD) of at least three independent experiments. The difference among measurement data was compared through one-way variance (ANOVA) and that between two different groups with the LSD test. Comparisons of multi-group ranked data were performed using the *Kruskal*–*Wallis* rank-sum test, and that of two different groups by the *Mann*–*Whitney* U test. A *p*-value of less than 0.05 was considered statistically significant.

## Results

### Model was successfully established

**Fig. 1:**
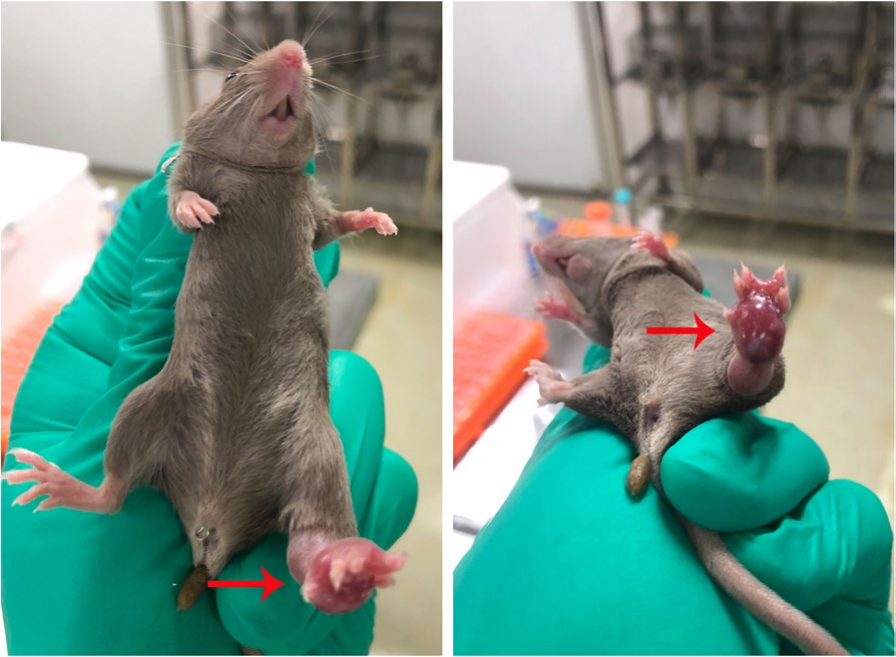
The degree of invasion has reached grade IV. Red arrows refer to the subcutaneous xenograft tumor of the left hind footpad. It was visible that the primary tumor invaded the epidermis, which became thinner and even a little broken

After inoculation of MFC cells on the 10th day, it was panic that primary tumors of most mice invaded muscle tissues diffusely. Some even invaded the epidermis. Judging from Table 2, the degree of invasion has reached grade III ~ IV (Fig. 1). Then, the tumorectomy (resection of primary tumors) was performed as described in the Methods section. On the next day after the tumorectomy, two mice died possibly from their physical factors that could not tolerate the resection of the left hind footpad, while the other mice lived well without infection.

Hence, the model was successfully established and divided into five groups randomly. As shown in Table 6, there was no significant difference in postoperative body weight and primary tumor weight among the five groups (*p* > 0.05).

**Table 6:** Postoperative body weight and primary tumor weight of 615 mouse models ($\overline{x }$±s)

Group	Postoperative weight (g)	Primary tumor weight (g)
Model control(*n* = 13)	24.07 ± 1.15	0.23 ± 0.05
5-Fu(*n* = 11)	24.81 ± 0.73	0.27 ± 0.08
Low-dose(*n* = 13)	24.49 ± 1.23	0.21 ± 0.06
Medium-dose(*n* = 13)	24.24 ± 1.36	0.26 ± 0.08
High-dose(*n* = 13)	24.31 ± 1.73	0.26 ± 0.10

### PBMF treatment showed resistance to body weight loss caused by tumor burden

During the PBMF treatment, 4, 3, 1 and 2 mice respectively died in the model control group, 5-Fu group, low- and high-dose groups. Dissection discovered that there were no abnormal pathological changes in the main organs. But scattered metastatic nodules could be seen on the lung tissue and the invasion of recurrence tumor at the incision was relatively serious, such as broken, bleeding, and necrosis. The remaining 53 mice survived until the end of treatment and the general condition was well.

**Fig. 2:**
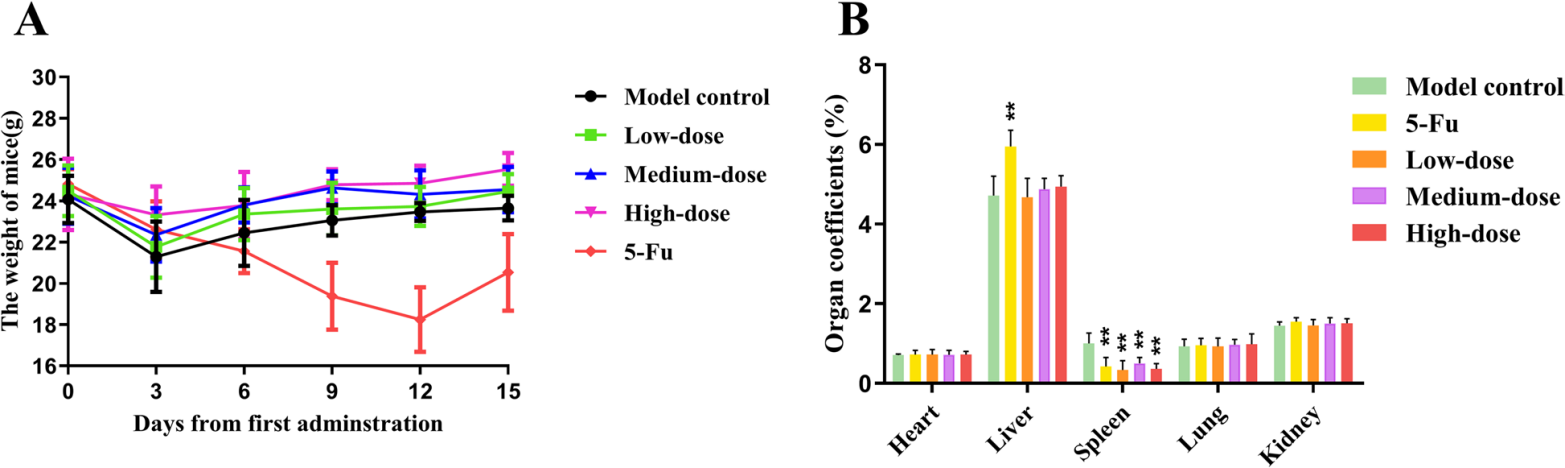
Effects of PMBF on body weights and organ coefficients. **A** Dynamic changes of the body weight in five groups during different treatments for 15 days. **B** Comparison of main organ coefficients. ^**^p < 0.01 vs. model control

The body weight of the five groups all decreased at the early stage of administration (Fig. 2A). From the third day of administration, except for the 5-Fu group, the weight in each group began to increase. The terminal weight of mice in the high-dose group was significantly increased but that in the 5-Fu group was significantly decreased, compared with the values in the model control group (*p* < 0.05). We speculate that the weight loss of the latter may attribute to side effects of the 5-Fu, a commonly used chemotherapeutic drug.

### Organ coefficients

Compared with the model control group, the organ coefficient of the spleen in the other four groups was markedly decreased and the coefficient of the liver in the 5-Fu group was significantly increased (*p* < 0.01) (Fig. 2B).

### PBMF treatment inhibited the growth of recurrence tumors

**Fig. 3:**
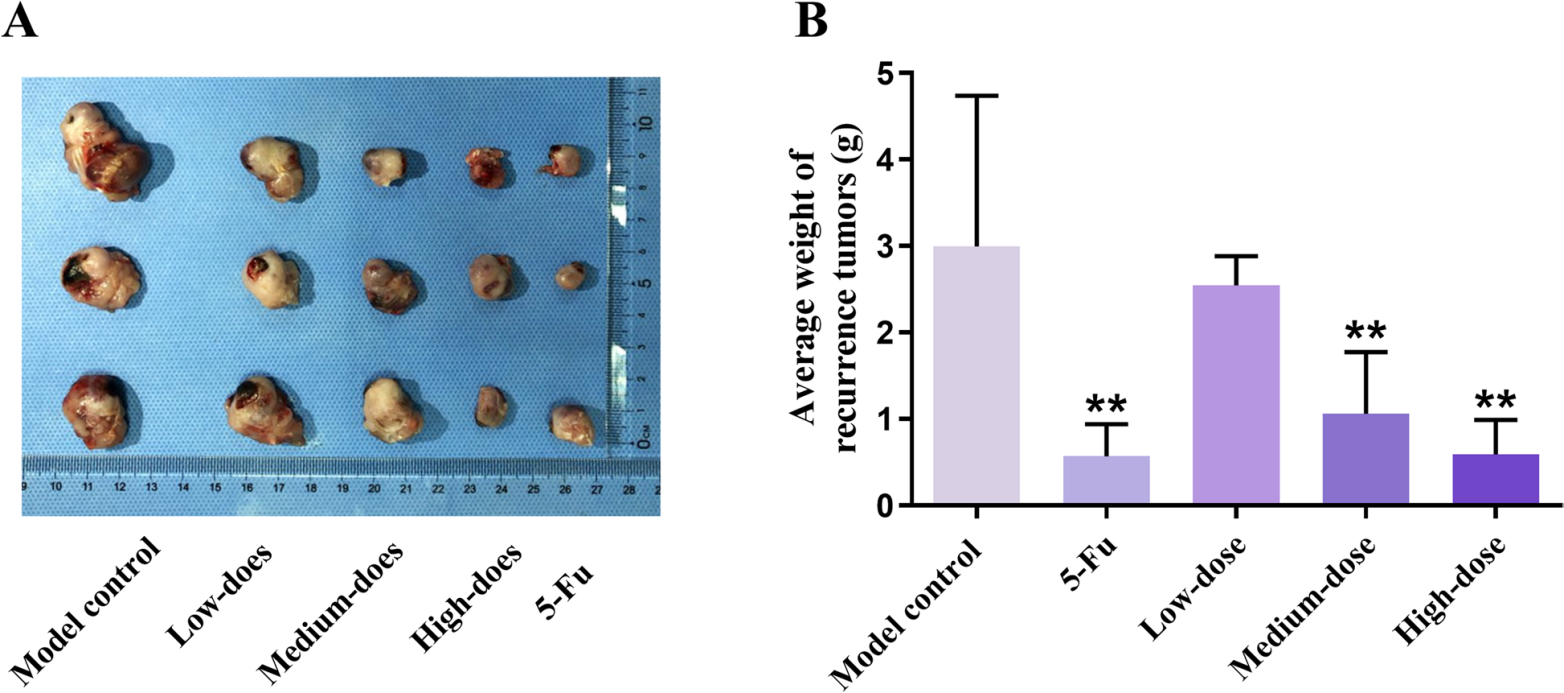
PBMF inhibited the recurrence tumor growth in 615 mice. **A** Representative photographs of recurrence tumors isolated from mice in five groups after sacrifice. **B** Average recurrence tumor weights in each group after 15 days’ administration. **p < 0.01 vs. model control

The size of recurrence tumors was presented in Fig. 3A. The average recurrence tumor weight of 5-Fu and PBMF-treated groups was lower than that of the model control group (*p* < 0.01). Moreover, 5-Fu, low-, medium-, and high-dose of PBMF inhibited the growth of recurrence tumors by 80.94, 14.96, 64.61, and 80.25% respectively (Table 7 and Fig. 3B).

**Table 7:** Recurrence tumor weight and tumor weight inhibition rate in 615 mice ($\overline{x }$±s)

Group	Recurrence tumor weight(g)	IR of recurrence tumor weight (%)
Model control	2.99 ± 1.74	—
5-Fu	0.57 ± 0.34^*^^*^	80.94
Low-dose	2.54 ± 0.34	14.96
Medium-dose	1.06 ± 0.72^*^^*^	64.61
High-dose	0.59 ± 0.40^**^	80.25

^**^p < 0.01 vs. model control

### PBMF reduced tumor recurrence and metastasis based on histological changes

#### H&E staining results of main organs

The histological morphology of the heart, liver, spleen, and kidney in each group was normal without pathological changes under the optical microscope.

#### H&E staining results of recurrence tumor tissues

**Fig. 4:**
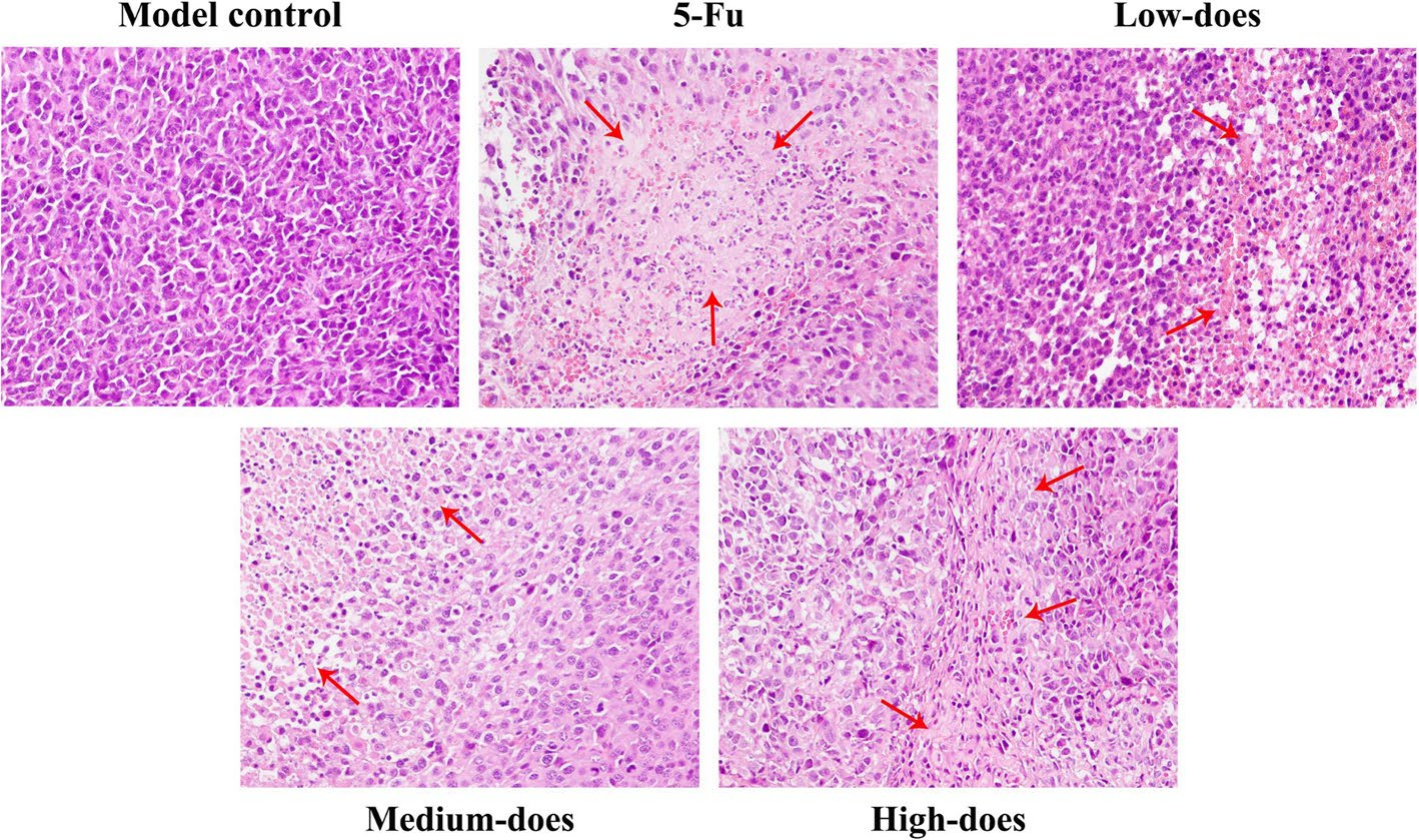
Histopathological changes of recurrence tumor tissues. Tumor necrosis areas and mitotic figures were determined by H&E staining and observed under an optical microscope (200 ×). Red arrows refer to the distinct necrosis areas

In recurrence tumor tissues, the model control group showed hyperchromatic and densely packed tumor cells with large nuclei, common mitotic figures, and less necrosis. By contrast, in 5-Fu and PBMF-treated groups, there were loosely arranged tumor cells, no mitotic figure, and more necrosis. 5-Fu group and the high-dose group were particularly obvious, in which large areas of necrosis could be seen with varying degrees of bleeding (Fig. 4).

#### H&E staining of lung tissues

**Fig. 5:**
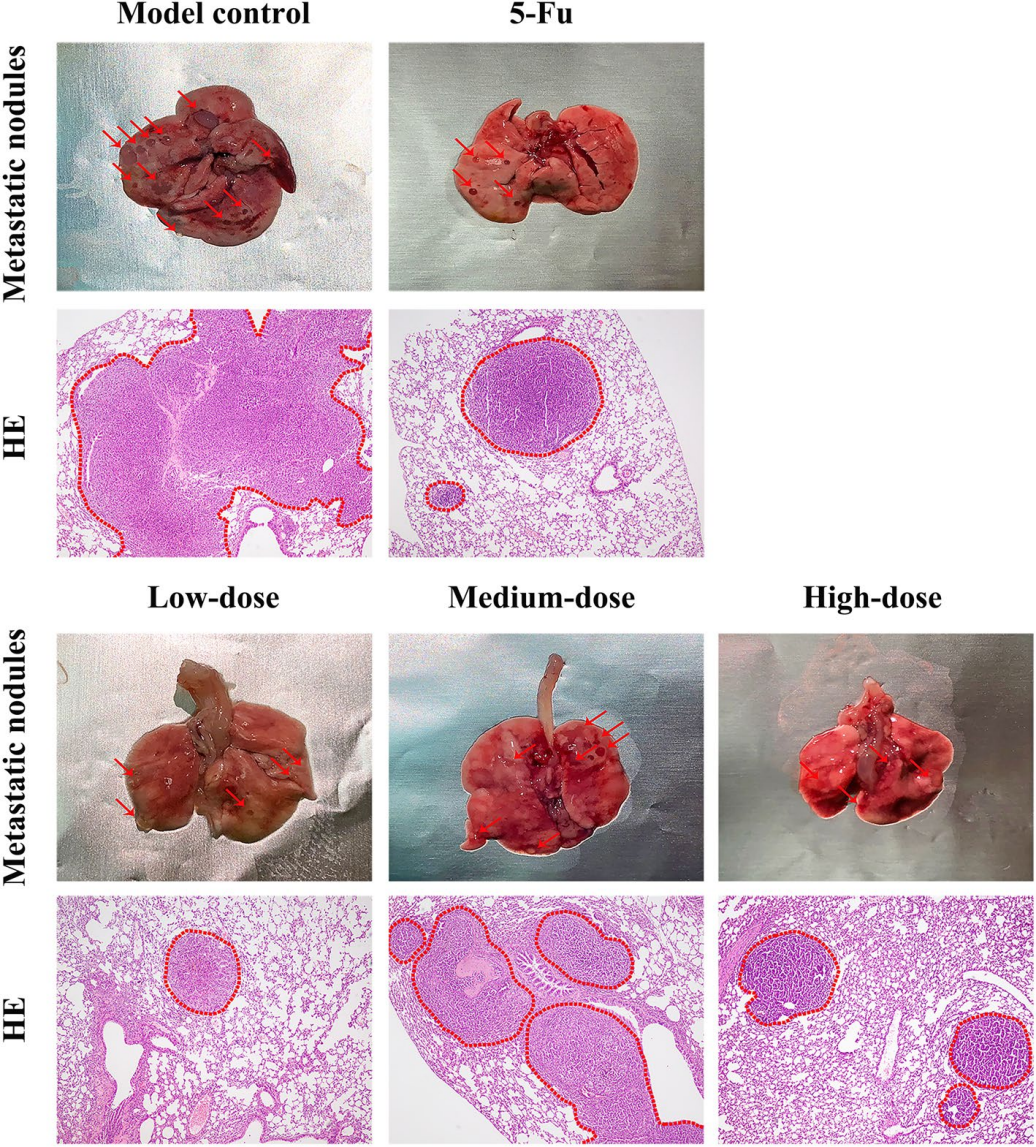
Lung metastatic nodules (naked eyes) and histopathological changes of lung tissues under low magnification (40 ×). Red arrows refer to the visible metastatic nodules on the surface of lung tissues. The red dotted line circled the metastases under low magnification (40 ×) after H&E staining

Metastatic nodules of different sizes were noticed on the surface of lung tissues in five groups. But in 5-Fu and PBMF-treated groups, the number and diameter of metastatic nodules were smaller. Metastases were large (more than 2 mm in diameter) and widely distributed in lung tissues of the model control group at low magnification, and some almost occupied one whole lung lobe (Fig. 5).

Lung metastasis scores of PBMF-treated groups were significantly lower than those of the model control group (*p* < 0.05). 5-Fu, low-, medium-, and high-dose PBMF inhibited lung metastasis by 24.94, 40.23, 44.53, and 43.71%, respectively (Table 8).

**Table 8:** Inhibition of lung metastasis after resection of xenograft tumor in 615 mice

Group	Number of mice	Grade of lung metastasis				Total score	Mean rank	IR of pulmonary metastasis (%)
		0	I	II	III			
Model control	9	0	1	3	5	22	40.22	-
5-Fu	8	2	1	3	2	13	30.19	24.94
Low-dose	12	4	5	1	2	13	24.04^*^	40.23
Medium-dose	13	7	2	1	3	13	22.31^*^	44.53
High-dose	11	5	3	1	2	11	22.64^*^	43.71

^*^p < 0.05 vs. model control

#### H&E staining of lymph node tissue

**Fig. 6:**
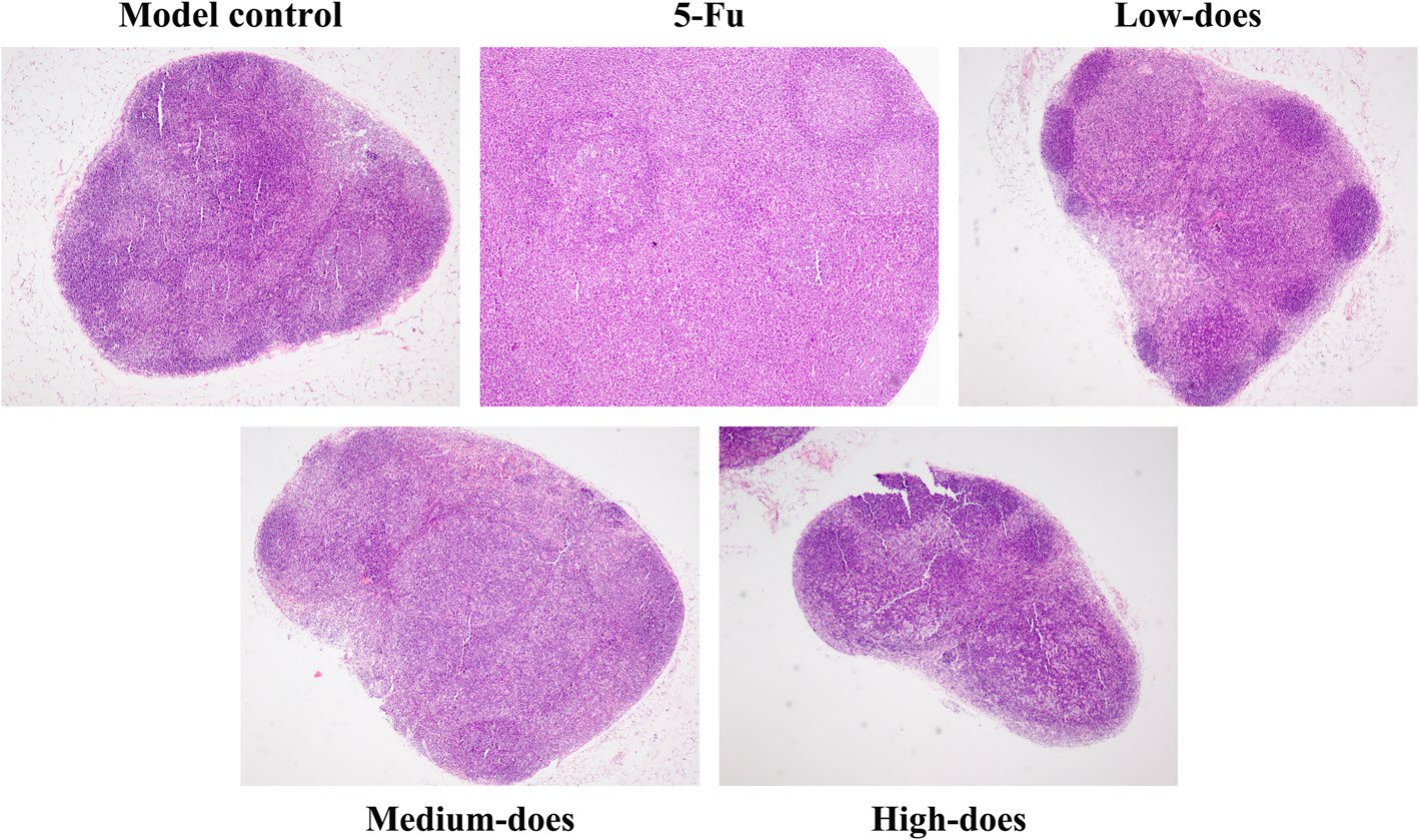
Histopathological changes of lymph node tissues under an optical microscope (40 ×). In the model control group, the lymph node tissues were invaded by numerous tumor cells (i.e., hyperchromatic cells with large nuclei) and clustered into masses to form extensive metastases. 5-Fu and PBMF-treated groups were better

H&E staining results showed the number of tumor cells in 5-Fu and PBMF-treated groups were decreased and scattered compared with that in the model control group. Only small metastases were seen in the marginal sinus of the lymph node tissue, and a few metastases appeared in the central sinus in 5-Fu and PBMF-treated groups (Fig. 6).

Lymph node metastasis scores of PBMF-treated groups were lower than those of the model control group, but without statistical significance (*p* > 0.05). 5-Fu, low-, medium-, and high-dose PBMF inhibited lymph node metastasis by 18.63, 19.17, 10.25, and 22.22%, respectively (Table 9).

**Table 9:** Inhibition of lymph node metastasis after resection of xenograft tumor in 615 mice

Group	Number of mice	Grade of lymph node metastasis					Total score	Mean rank	IR of lymph node metastasis (%)
		0	I	II	III	IV			
Model control	9	1	2	0	1	5	25	31.50	-
5-Fu	8	0	1	4	2	1	19	25.63	18.63
Low-dose	12	1	2	5	0	4	28	25.46	19.17
Medium-dose	13	1	0	6	3	3	33	28.27	10.25
High-dose	11	2	0	5	2	2	24	24.50	22.22

### mRNA expressions of β-catenin, GSK-3β, E-cadherin, Vimentin, VEGF-C/D, and VEGFR-2/3 genes

#### mRNA expression of β-catenin, GSK-3β, E-cadherin, Vimentin, VEGF-C/D, and VEGFR-2/3 genes in recurrence tumors

**Fig. 7:**
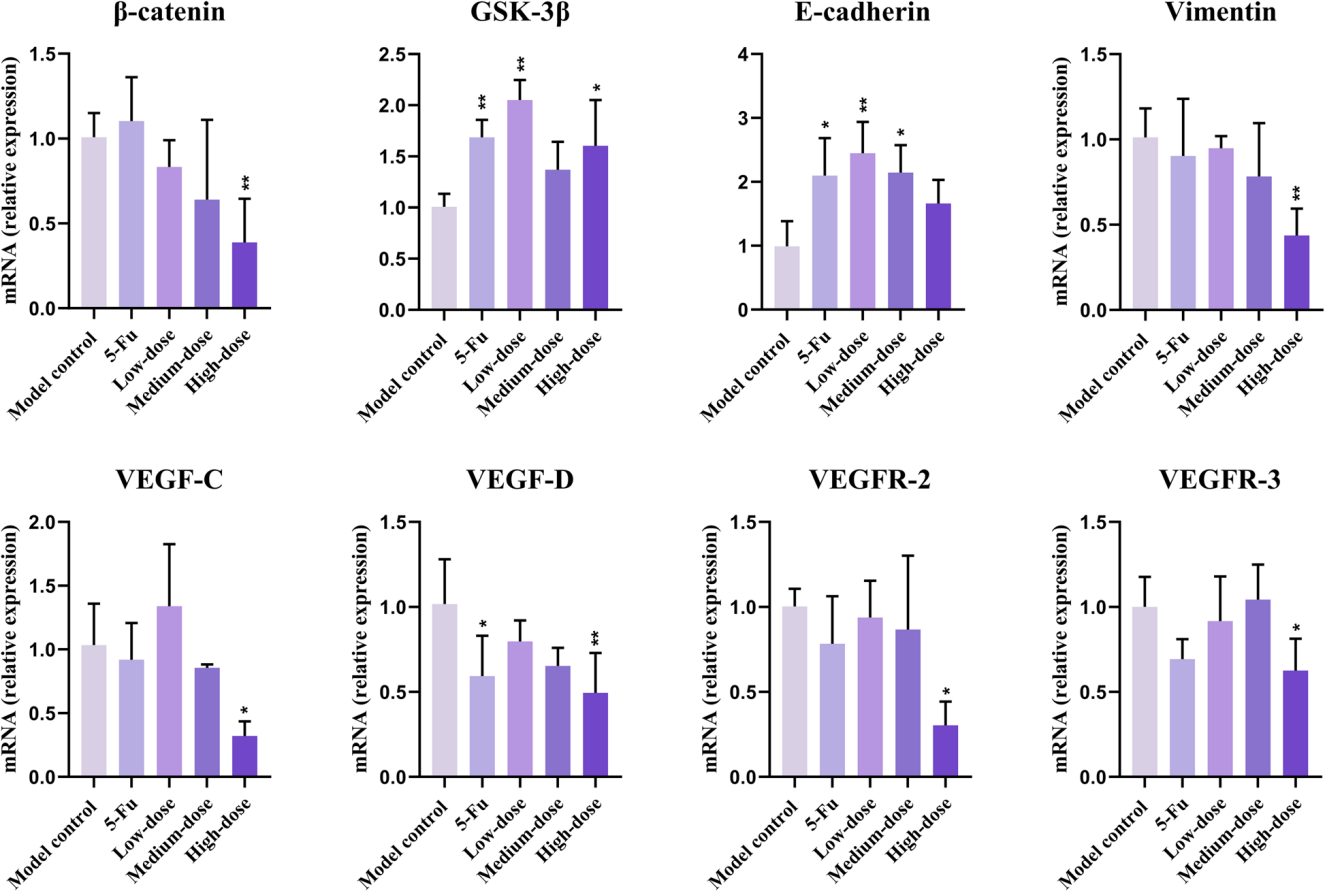
mRNA expressions of specific genes involved in EMT, Wnt/β-catenin pathway, and lymphangiogenesis in recurrence tumors. Results were presented as mean ± SD. ^*^p < 0.05 vs. model control; ^**^p < 0.01 vs. model control

The mRNA expression of β-catenin was markedly lower in the high-dose group and GSK-3β was higher in 5-Fu, low- and high-dose groups (*p* < 0.05), compared with the values in the model groups. mRNA expression of E-cadherin was up-regulated in 5-Fu, low-and medium-dose groups, and Vimentin was significantly down-regulated in the high-dose group (*p* < 0.05) (Fig. 7).

Compared with the model control group, mRNA levels of VEGF-C, VEGFR-2, and VEGFR-3 of recurrence tumor tissues were significantly decreased in the high-dose group (*p* < 0.05). mRNA levels of VEGF-D in the 5-Fu group and the high-dose group were lower (*p* < 0.05), compared with the values in the model group.

#### mRNA expression of VEGF-C/D and VEGFR-2/3 gene in lung tissues

**Fig. 8:**
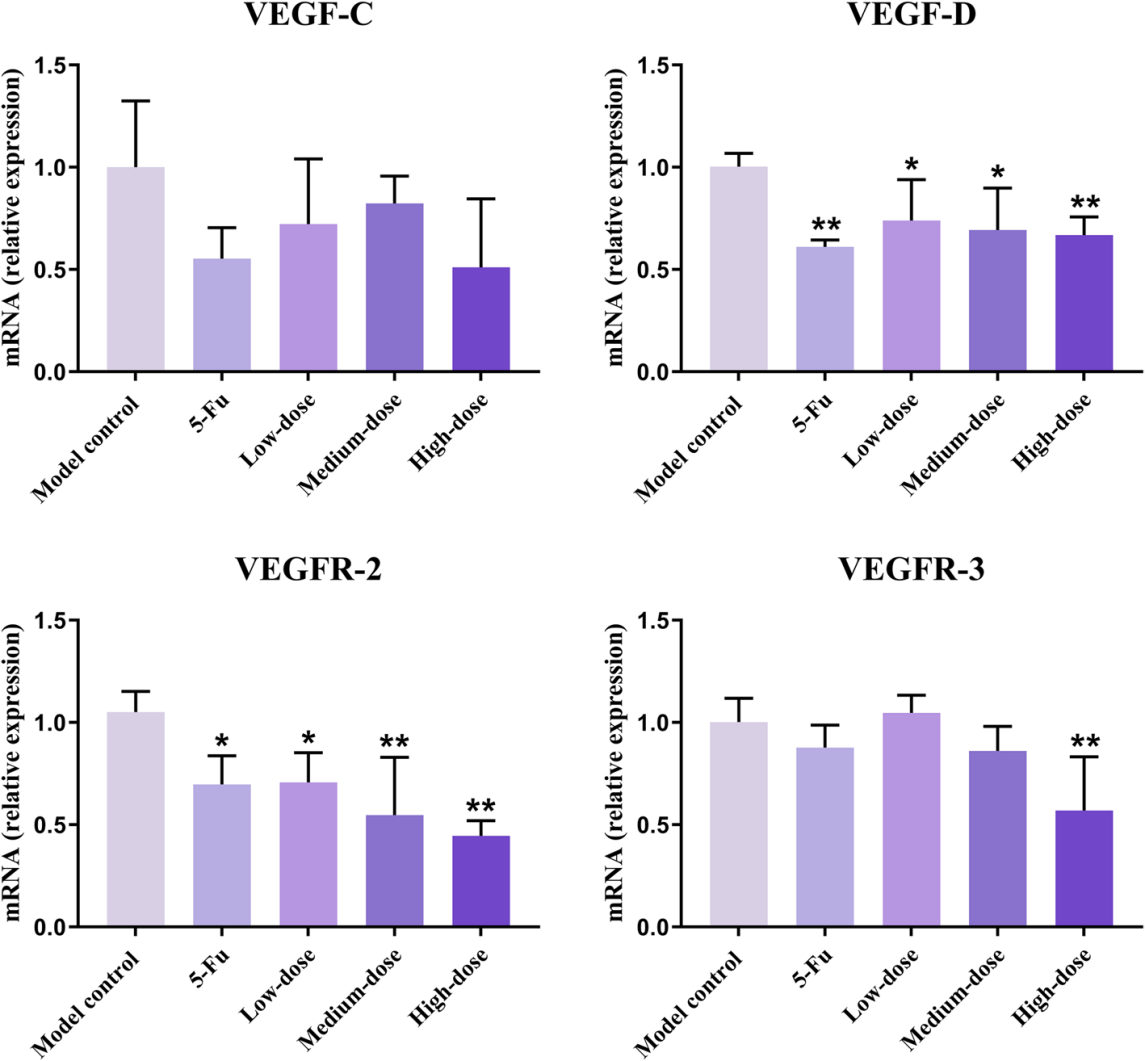
mRNA expression of VEGF-C, VEGF-D. VEGFR-2, VEGFR-3 genes in lung tissues. Results were presented as mean ± SD. ^*^p < 0.05 vs. model control; ^**^p < 0.01 vs. model control

In lung tissues, 5-Fu and PBMF treatments markedly down-regulated the mRNA levels of VEGF-D and VEGFR-2 (*p* < 0.05). VEGFR-3 was also significantly lower in the high-dose group (*p* < 0.05). Moreover, mRNA expression of VEGF-C in other groups presented a downward trend but the difference was not statistically significant compared with the value in the model control group (*p* > 0.05) (Fig. 8).

### Western Blot of β-catenin, p-β-catenin (Ser33/37/Thr41), GSK-3β, p-GSK-3β (Ser9), E-cadherin, and Vimentin in recurrence tumors

**Fig. 9:**
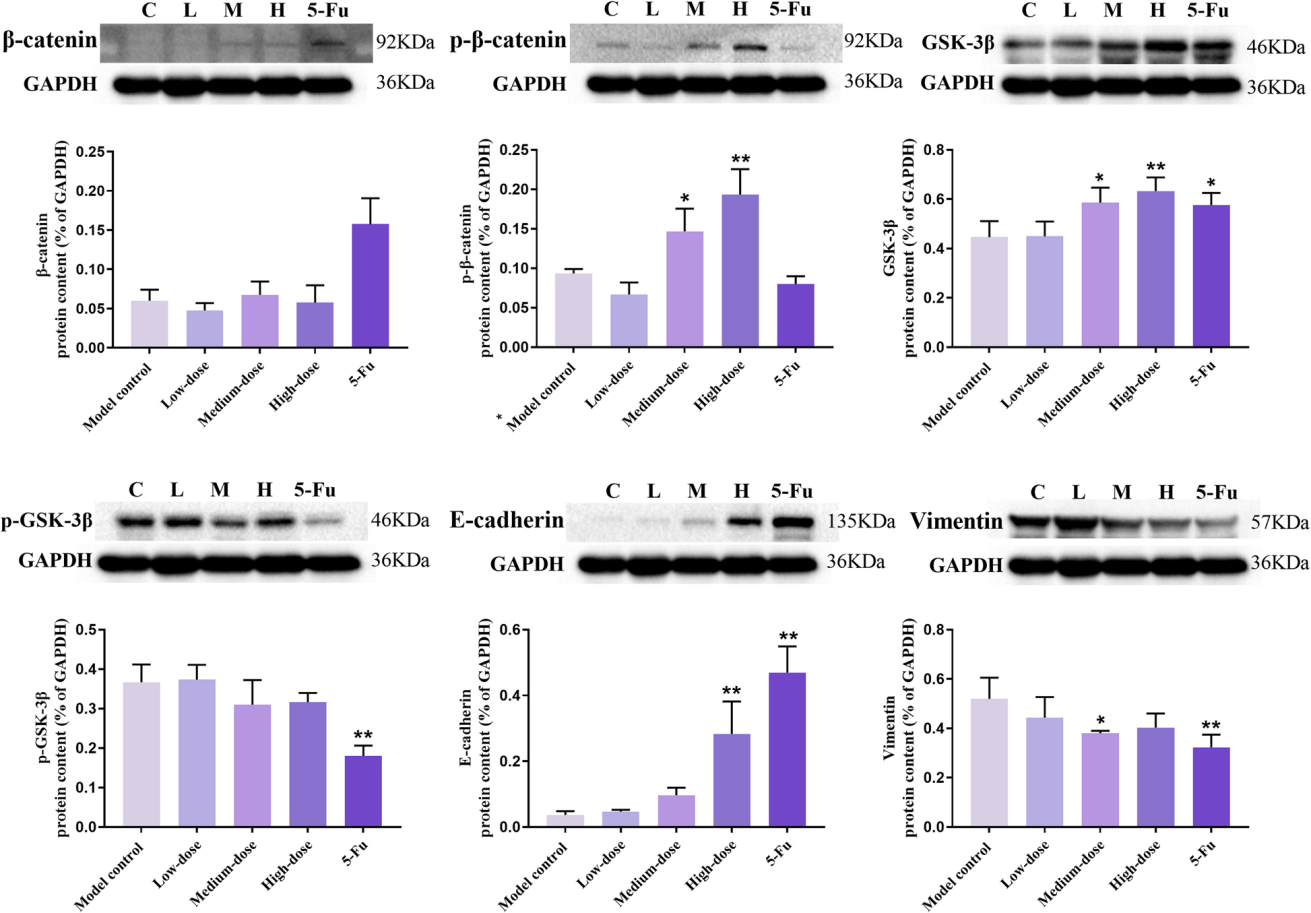
Western blot showed the cropped gels and the protein contents. The protein was related to Wnt/β-catenin and EMT signaling pathway in recurrence tumor tissues. Results were presented as mean ± SD. ^*^p < 0.05 vs. model control; ^**^p < 0.01 vs. model control. The original, uncropped, and replicated blots are presented in Supplementary Fig. 1

**Fig. 10:**
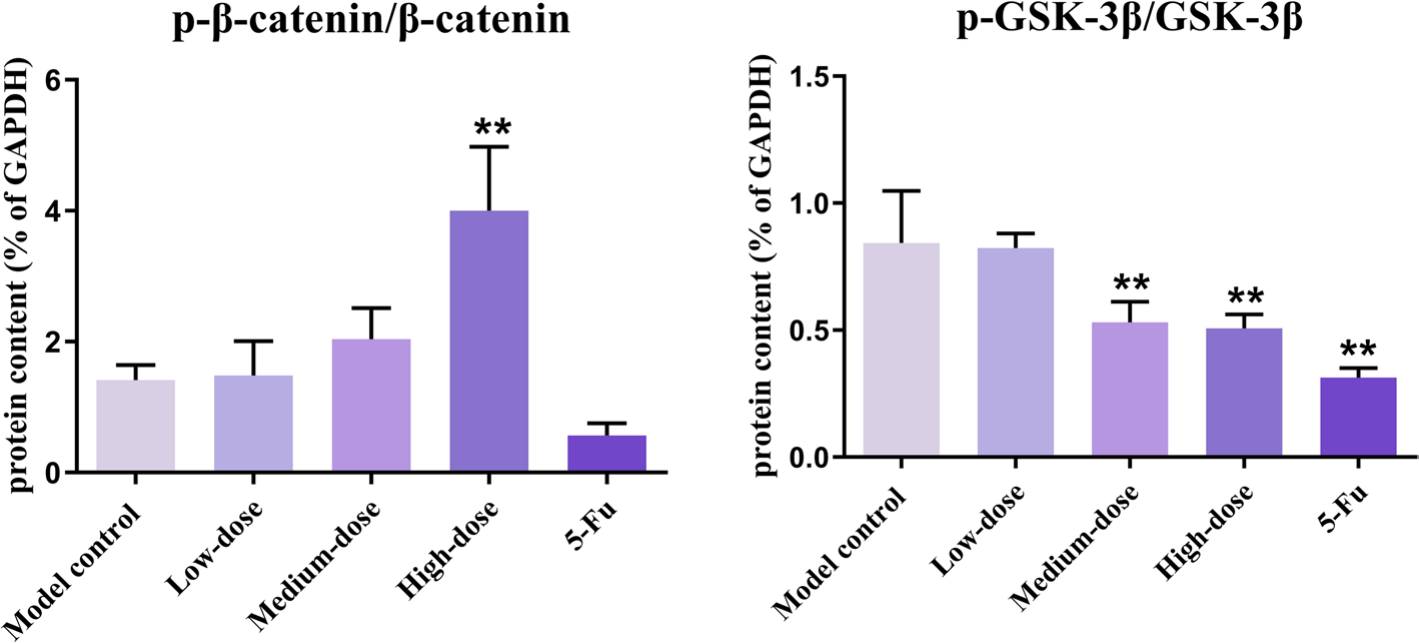
The ratios of p-β-catenin (Ser33/37/Thr41) to β-catenin and p-GSK-3β (Ser9) to GSK-3β in recurrence tumors. Results were presented as mean ± SD. ^**^p < 0.01 vs. model control

There was no significant difference in the expression of β-catenin protein among groups (*p* > 0.05). However, in comparison to the model control group, p-β-catenin (Ser33/37/Thr41) protein contents in middle- and high-dose groups dramatically increased (*p* < 0.05). As well as the ratio of p-β-catenin (Ser33/37/Thr41)/β-catenin in the high-dose group was far higher (*p* < 0.01). GSK-3β protein contents in 5-Fu, middle- and high-dose groups were markedly elevated, and p-GSK-3β (Ser9) in the 5-Fu group was lower than those in the model control group. Moreover, the ratio of p-GSK-3β (Ser9)/GSK-3β in the 5-Fu group, middle- and high-dose groups was much lower (*p* < 0.01) (Figs. 9 and 10).

Compared with the values in the model control group, the protein levels of E-cadherin in 5-Fu and high-dose groups were significantly raised. By contrast, these of Vimentin in 5-Fu and medium-dose groups were pronouncedly decreased (*p* < 0.05) (Fig. 9).

### Immunohistochemistry staining of LYVE-1, VEGF-C/D, and VEGFR-3

#### LVD in recurrence tumors and lung tissues

**Fig. 11:**
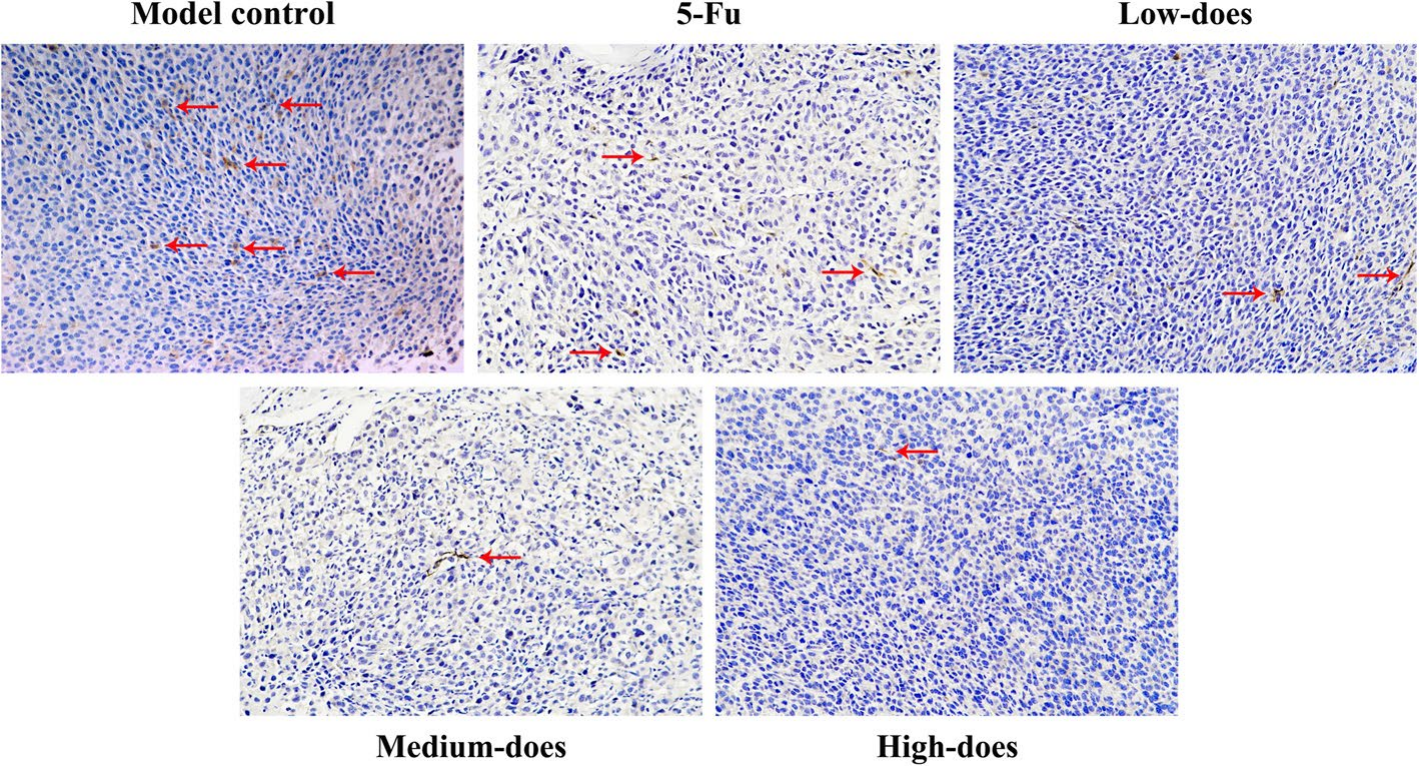
Immunohistochemistry results of LVD in recurrence tumor tissues (200 ×). The area indicated by the red arrow is a representative LVD marked by LYVE-1

**Fig. 12:**
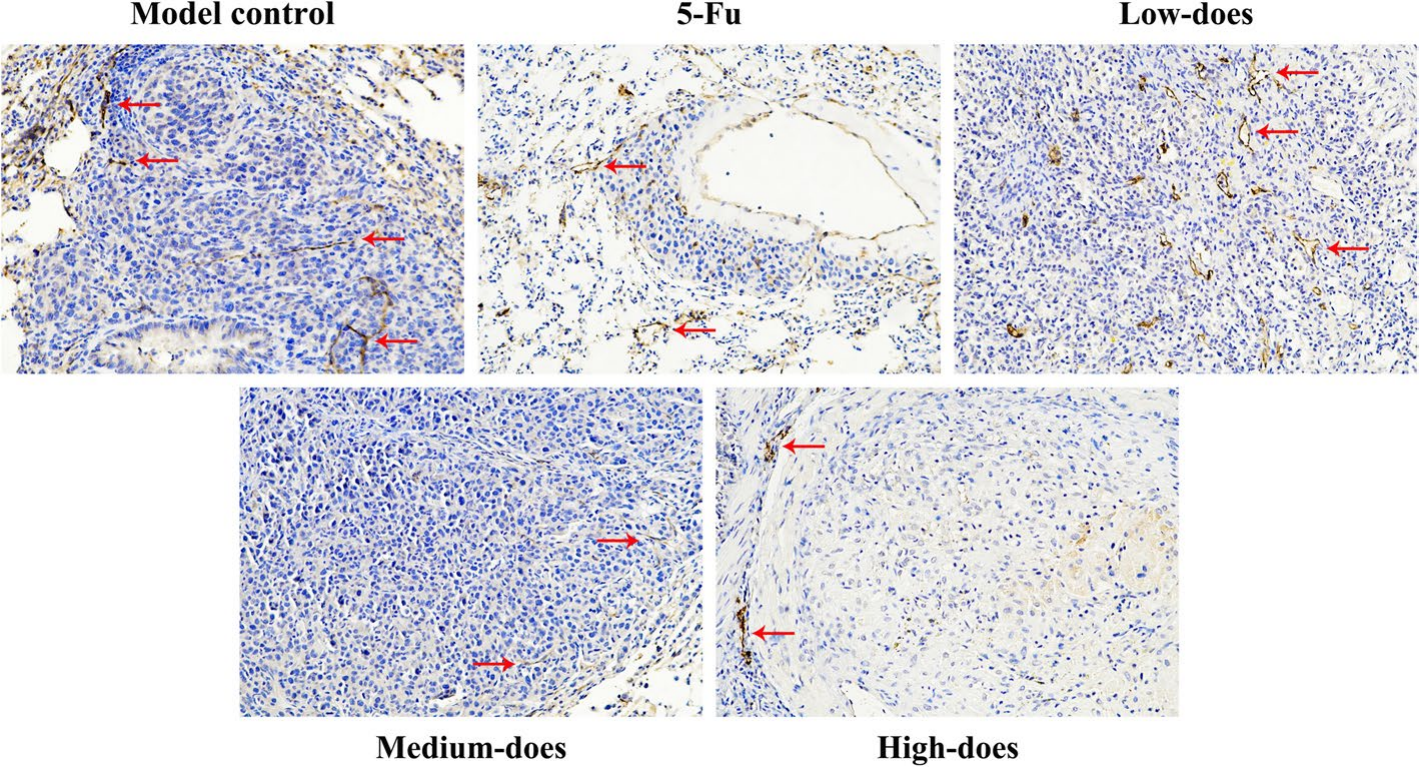
Immunohistochemistry results of LVD in lung tissues (200 ×). The area indicated by the red arrow is a representative LVD marked by LYVE-1

**Fig. 13:**
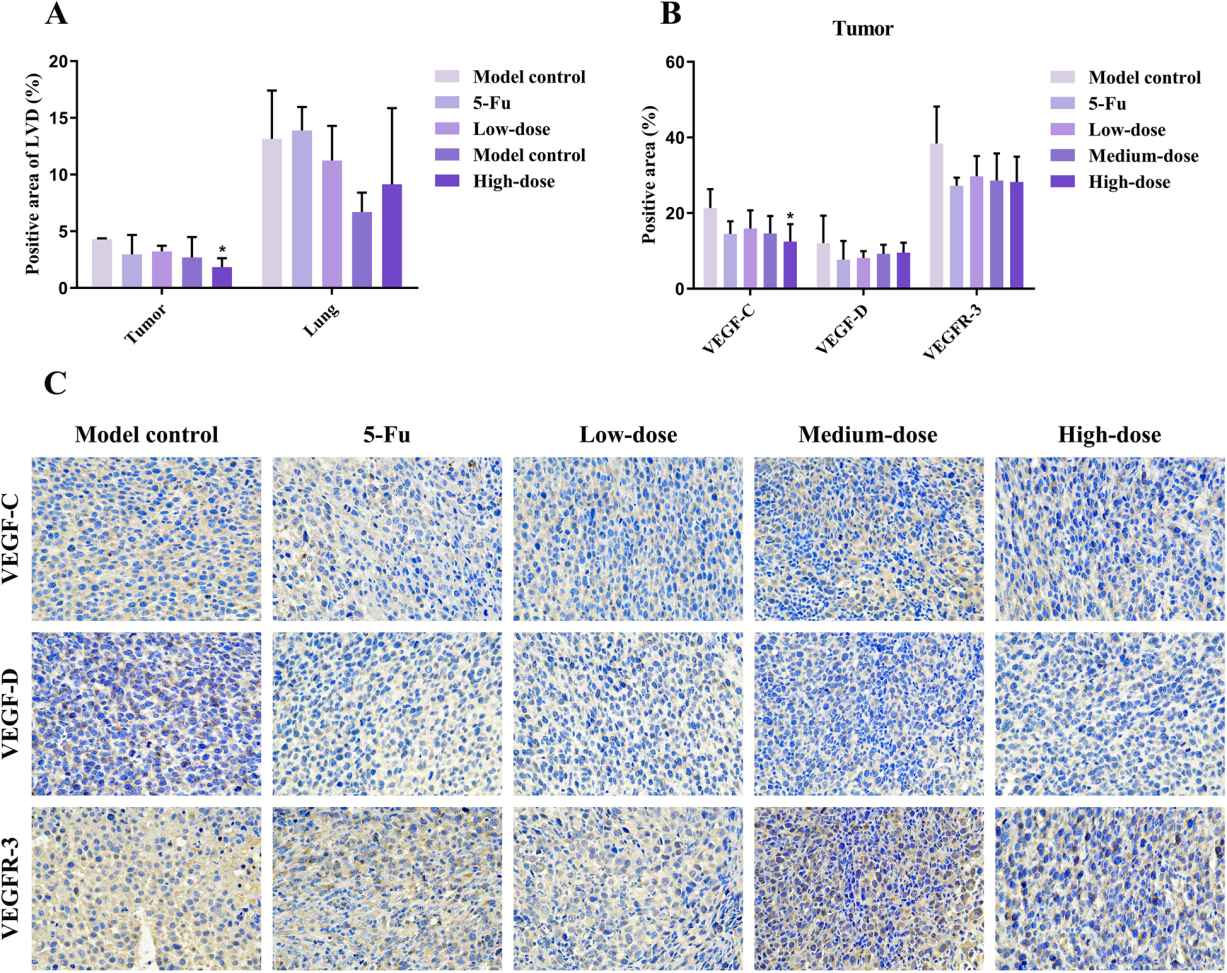
Effects of PBMF on LVD and related proteins in recurrence tumor and lung tissues. **A** Comparison of LVD in recurrence tumor and lung tissues. **B** VEGF-C, VEGF-D, and VEGFR-3 protein levers in recurrence tumor tissues. Results were presented as mean ± SD. ^*^p < 0.05 vs. model control (C) Immunohistochemical results of VEGF-C, VEGF-D, and VEGFR-3 proteins in recurrence tumor tissues (400 ×). Target proteins were mainly expressed in the cytomembrane and cytoplasm. The positive staining was pale yellow or brown granular

As shown in Figs. 11 and 12, LVD of PBMF-treated groups showed a downward trend in recurrence tumor and lung tissues. And LVD of recurrence tumor tissues was significantly lower in the high-dose group compared with that in the model control group (*p* < 0.05) (Fig. 13A).

#### Protein expression levels of VEGF-C, VEGF-D, and VEGFR-3 in recurrence tumor tissues

VEGF-C/D and VEGFR-3 are important regulators of lymphangiogenesis. It can be seen from Fig. 13B that the level of VEGF-C in the high-dose group was significantly higher (*p* < 0.05). Compared with the model control group, VEGF-C in other groups, VEGF-D and VEGFR-3 in PBMF-treated groups presented a downward trend with no statistical difference (*p* > 0.05).

## Discussion

PBMF has been previously proved to have certain anti-tumor effects in a subcutaneous xenograft tumor model of nude mice, including inhibiting tumor cell proliferation and inducing apoptosis [27]. On this basis, our study is devoted to deeply exploring the effects of PBMF on postoperative recurrence and distant metastasis, while its underlying mechanisms are further elucidated. The above results indicated that PBMF could inhibit the growth of local recurrence tumor and lung metastasis in GC, suppress the Wnt/β-catenin signaling pathway, and reverse the EMT process. The lymphangiogenesis was also restrained presumably related to the down-regulation of VEGF-C/D and VEGFR-2/3.

The 615 mouse model of postoperative recurrence and metastasis was successfully established by using the homologous tumor cell. Referred to the methods recommended by Gao et al. The MFC cell originated from 615 mice, which was a cell line with a high potential for spontaneous lung metastasis, and the success rate of xenograft was as high as 100% in inbred 615 mice. At the same time, Selecting the footpad of the left hind leg of mice as the subcutaneous inoculation site was the best way to obtain lymphatic metastasis. Therefore, the model could better simulate the metastasis model of human GC, which was mainly with lung metastasis and combined with lymph node metastasis [28, 30]. The model has many advantages, such as simple operation, less damage to mice, rapid postoperative recovery, less prone to wound infection, and so on. Meanwhile, the postoperative recurrence and metastasis model established by this method was similar to that of patients with AGC after surgical resection.

After 15 days’ administration, the recurrence tumor weight of the 5-Fu group, medium- and high-dose groups was significantly decreased, suggesting that PBMF effectively inhibited the growth of recurrence tumor after tumor resection. Moreover, the IR of recurrence tumor weight in the high-dose group (80.25%) was close to that of the 5-Fu group (80.94%). The higher terminal body weight in the high-dose group indicated that PBMF could improve the quality of life in later life. H&E staining results of recurrence tumor tissues showed that tumor cells in PBMF-treated groups were more loosely arranged, had fewer or no mitotic figures, and there was more necrosis than the model control group, especially in the 5-Fu group and high-dose group, which further confirmed the anti-tumor effect of PBMF.

It was found that the organ coefficient of the liver in the 5-Fu group was significantly higher than that in the model control group. But the organ coefficients of the spleen in 5-Fu and PBMF-treated groups were much lower. The liver is the main place for drug metabolism, including 5-Fu [31]. And 5-Fu group was stimulated by positive drugs resulting in physiological enlargement of the liver, which was the side effect of 5-Fu. Abnormal enlargement of the spleen occurred only in the model control mice. It was speculated that the influence of pathological conditions such as malignant invasive growth and distant metastasis of recurrence tumor over-activated immune system. Under the intervention of 5-Fu and different doses of PBMF, the enlargement was significantly alleviated.

Distant metastasis of GC was one of the most important causes of poor prognosis. Tumor metastatic nodules of lung tissue were evident through visual observation. Especially in the model control group, metastatic nodules were in dense distribution and greater number. At the same time, H&E staining of lung tissues showed that the number and diameter of lung metastases in the PBMF-treated group decreased dramatically and lung metastasis scores were lower. Lymph node metastasis scores of 5-Fu and PBMF-treated groups presented a downward trend. In conclusion, PBMF had a certain inhibitory effect on lung metastasis and lymph node metastasis after tumor resection in inbred 615 mice.

**Fig. 14:**
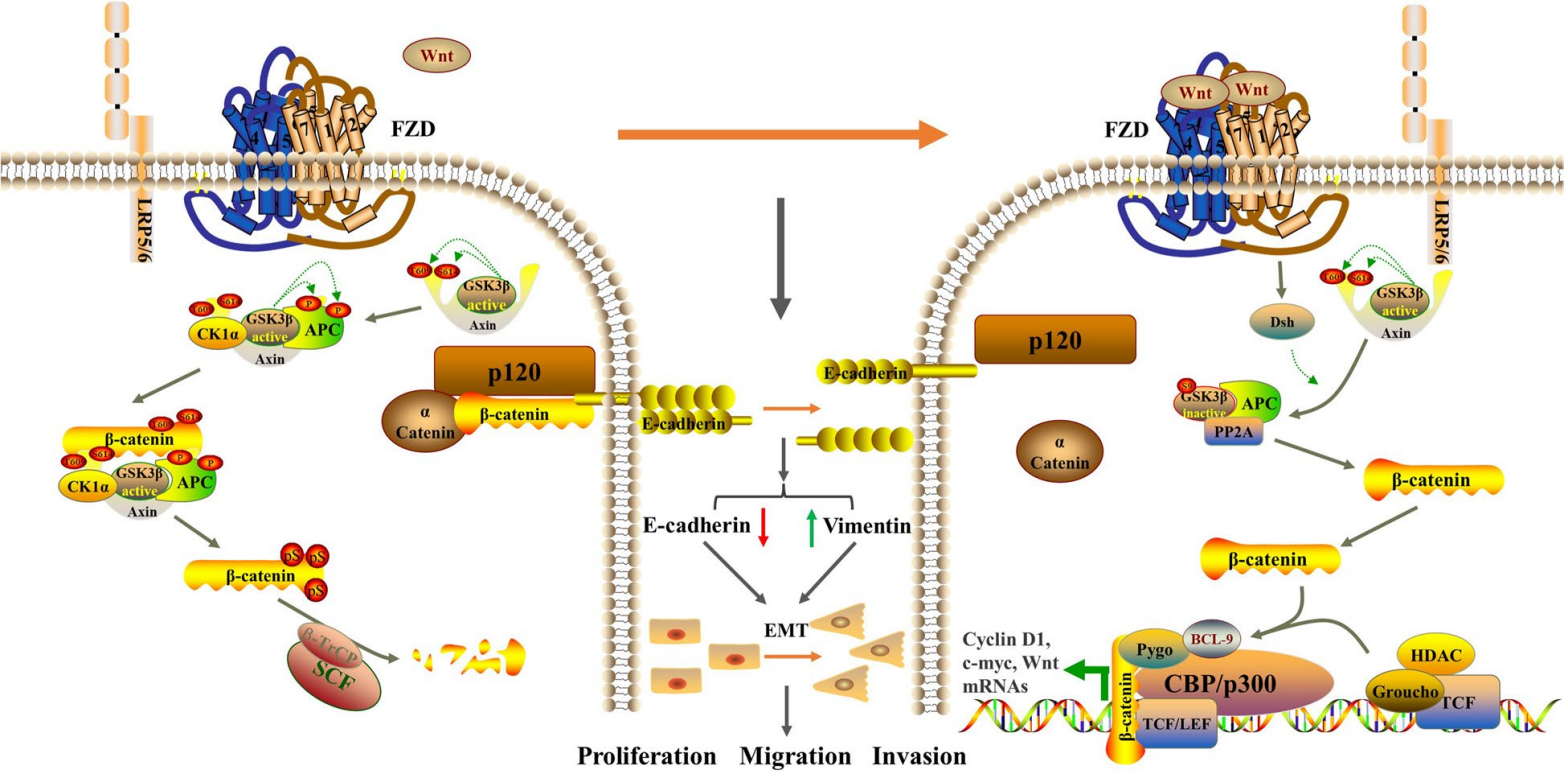
The regulatory process of Wnt/β-catenin signaling pathway on EMT

EMT and Wnt/β-catenin are both crucial signaling pathways in tumorigenesis and development. The main characteristics of EMT are mesenchymal cell markers such as N-cadherin and Vimentin are up-regulated, and epithelial cell markers such as E-cadherin and cytokeratin are down-regulated. Dysregulation of the canonical Wnt/β-catenin signaling pathway can induce EMT [32]. The regulatory process is shown in Fig. 14, which mainly involves the stability of proto-oncogene β-catenin. So β-catenin and its inhibitor GSK-3β are regarded as the central regulatory factors of the Wnt/β-catenin signaling pathway [33, 34]. In this study, E-cadherin showed higher lever both in mRNA and protein expression.β-catenin and Vimentin mRNA levels were lower in the high-dose group, and GSK-3β protein level was enhanced consistently in the high-dose group, indicating that Wnt/β-catenin and EMT signaling pathway was both suppressed by PBMF.

The activity of GSK3β is mainly regulated by its Ser9 phosphorylation [35]. Without Wnt signaling, phosphorylation of β-catenin by GSK-3β at Ser33/37/Thr41 results in ubiquitination and degradation of cytoplasmic β-catenin [36]. The phosphorylation of total β-catenin and total GSK-3β protein converted to p-β-catenin (Ser33/37/Thr41) and p-GSK-3β (Ser9) respectively, which inhibited the activity of their original protein. Therefore, increasing the phosphorylation level of β-catenin and reducing the phosphorylation level of GSK-3β is essential for inhibiting the activation of the Wnt/β-catenin pathway. Although there was no difference in β-catenin protein expression among different groups, the p-β-catenin (Ser33/37/Thr41) protein expression, and p-β-catenin (Ser33/37/Thr41)/β-catenin ratio were significantly higher in the high-dose group. The p-GSK-3β (Ser9)/GSK-3β ratio was correspondingly decreased. Based on these results, it is suggested that PBMF may reverse EMT mediated by inhibiting the Wnt/β-catenin signaling pathway.

Studies have confirmed that the recurrence and metastasis of GC are closely related to lymphangiogenesis. Early gastric tumor cells are prone to local or distant dissemination through lymphatic metastasis, which severely affects the clinical efficacy [37, 38]. So lymphangiogenesis is regarded as a key indicator for evaluating the metastasis process and predicting the prognosis of GC patients [23, 39]. We have assessed LVD labeled by LYVE-1 to reflect the lymphangiogenesis. The immunohistochemistry results showed that the LVD of PBMF-treated groups was in a downward trend and the high-dose group was markedly lower in recurrence tumors. Therefore, PBMF has a certain inhibitory effect on lymphangiogenesis in recurrent tumors and lung tissues.

VEGF-A/B/C/D and VEGFR-1/2/3 are members of the VEGF family, which mainly regulate the growth of vascular endothelial cells and lymphatic endothelial cells [40]. VEGF-C and VEGF-D can bind to the specific VEGFR-3 on the surface of lymphatic endothelial cells to form a complex and activate the VEGF-C/D-VEGFR-3 signaling cascade [41–43], which plays a important role in lymphangiogenesis [38, 44–46]. VEGFR-2 participates in the regulation of angiogenesis by binding to VEGF-A. However, Karkkainen et al. found that VEGFR-2 in lymphatic endothelium could also be activated by VEGF-C/D and participate in the regulation of lymphangiogenesis [47]. Activated VEGFR-2 can likewise combine with VEGFR-3 to form a heterodimer, which promotes the utilization of phosphorylation sites and finally accelerates the VEGF-C/D-VEGFR-3 signaling cascade [48]. In the present study, PBMF decreased mRNA expressions of VEGF-C, VEGF-D, VEGFR-2, and VEGFR-3. Meanwhile, the immunohistochemistry results demonstrated that VEGF-C protein was at a lower level consistently. Hence, it is suggested that PBMF seems to possess the anti-lymphatic metastasis effect by inhibiting the activation of the VEGF-C/D-VEGFR-2/3 signaling cascade, and finally suppressing lymphangiogenesis.

In our previous research, we studied the effect of PBMF on proliferation and apoptosis. The protein expression of Ki-67 and the mRNA expression of Cyclin D1 were significantly reduced after PBMF treatment. The percentage of TUNEL-positive cells showed that PBMF significantly induced tumor apoptosis. Based on the results of the first phase, we continued to explore the effect of PBMF on tumor recurrence and metastasis. Furthermore, β-catenin and GSK-3β of the Wnt/β-catenin signaling pathway are also associated with cell proliferation and apoptosis, so their expression levels can still reflect proliferation or apoptosis to a certain extent. Activation of VEGFR-3 also promotes lymphatic endothelial cell proliferation and inhibits apoptosis. Our findings in the pathway support the role of PBMF in proliferation or apoptosis. *Coix* seed, *L. edodes*, *A. officinalis* L., *H. cordata,* and other components are both medicinal and edible plants with certain anti-tumor effects. As it turned out, PBMF possesses the characteristics of anti- recurrence and anti-metastasis in the GC mouse model.

## Conclusions

In summary, the postoperative recurrence and metastasis model of GC in inbred 615 mice was successfully established. The PBMF exhibited beneficial effects on inhibiting the growth of postoperative local recurrence tumor and lung metastasis in GC. While the potential mechanisms could be the reversion of EMT mediated by suppressing the Wnt/β-catenin signaling pathway, thereby delaying the recurrence of GC after resection. Meanwhile, PBMF could inhibit lymphangiogenesis by suppressing the activation of the VEGF-C/D-VEGFR-3 signaling cascade.

## Supplementary Information

**Additional file 1.**

## Data Availability

All data generated or analyzed during this study are included in this published article.

## Abbreviations

AGC: Advanced gastric cancer
bFGF: Basic fibroblast growth factor
DAB: 3′-Diaminobenzidine
EMT: Epithelial-mesenchymal transition
GC: Gastric cancer
GSK-3β: Glycogen synthase kinase-3β
H&E: Hematoxylin and eosin
IARC: International Agency for Research on Cancer
IOD: Integrated optical density
IR: Inhibition rate
LP1: Latcripin 1
LVD: Lymphatic vessel density
LYVE-1: Lymphatic vessel endothelial hyaluronic acid receptor-1
MFC: Mouse forestomach carcinoma
ANOVA: One-way variance
PBMF: Plant-based medicinal food
RT-qPCR: Real-time Quantitative Polymerase Chain Reaction
SD: Standard deviation
TCM: Traditional Chinese Medicine
TUNEL: TdT-mediated dUTP Nick-End Labeling
VEGF-C/D: Vascular endothelial growth factor-C/D
VEGFR-2/3: Vascular endothelial growth factor receptor-2/3

## Declarations

### Other statements

The study was carried out in compliance with the ARRIVE guidelines (Animal Research: Reporting of In Vivo Experiments). Experimental research on plants complied with the IUCN Policy Statement on Research Involving Species at Risk of Extinction and the Convention on the Trade in Endangered Species of Wild Fauna and Flora.

All medicinal plants are purchased commercially from Bozhou Traditional Chinese Medicine Market (Bozhou China) by Jiangxi Gongqing Jiangzhong Dietary Therapy Technology Ltd (Jiujiang China). And the collection of seeds or plants was not involved.
