# Supplementary material for: Effects of plant-based medicinal food on postoperative recurrence and lung metastasis of gastric cancer regulated by Wnt/β-catenin-EMT signaling pathway and VEGF-C/D-VEGFR-3 cascade in a mouse model
Source: BMC Complement Med Ther. 2022 Sep 2;22:233. doi: 10.1186/s12906-022-03703-0 (PMC9438347; doi:10.1186/s12906-022-03703-0)
Supplement: Supplementary file 1 — Additional file 1. [file 12906_2022_3703_MOESM1_ESM.pdf]

β-catenin
92 KDa

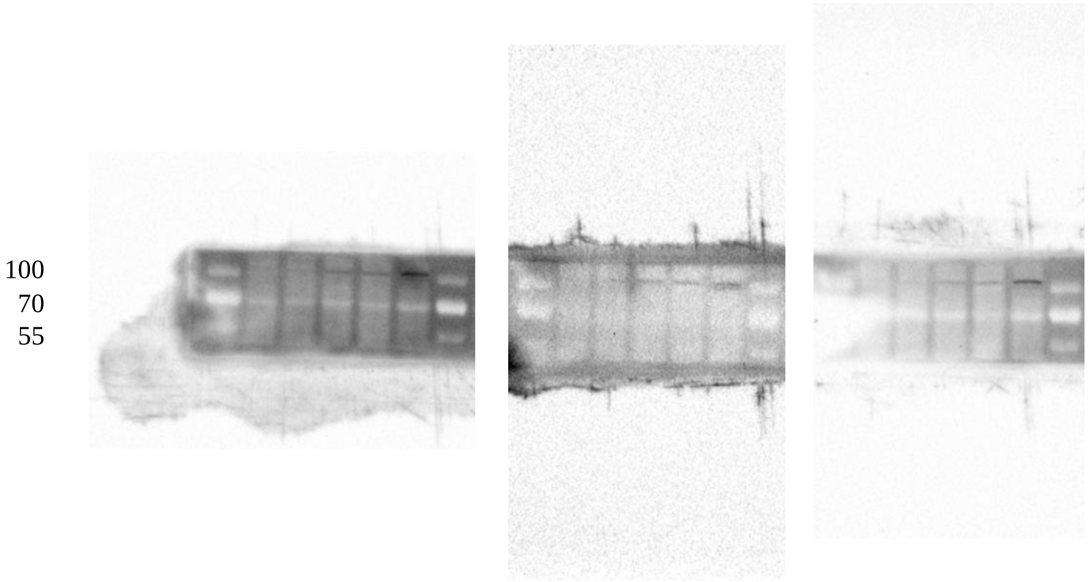

p-β-catenin
92 KDa

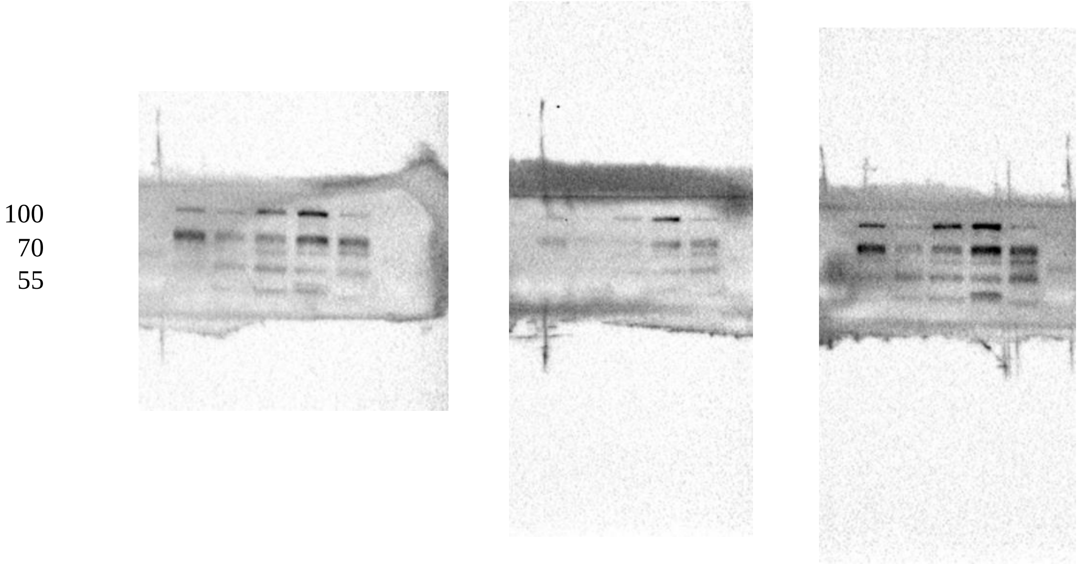

GSK-3β
46 KDa

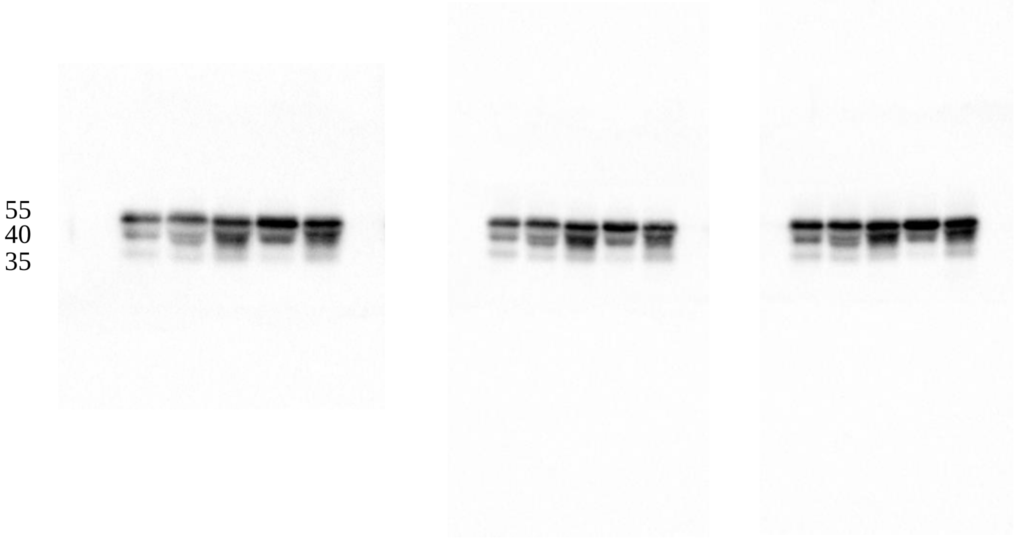

p-GSK-3β
46 KDa

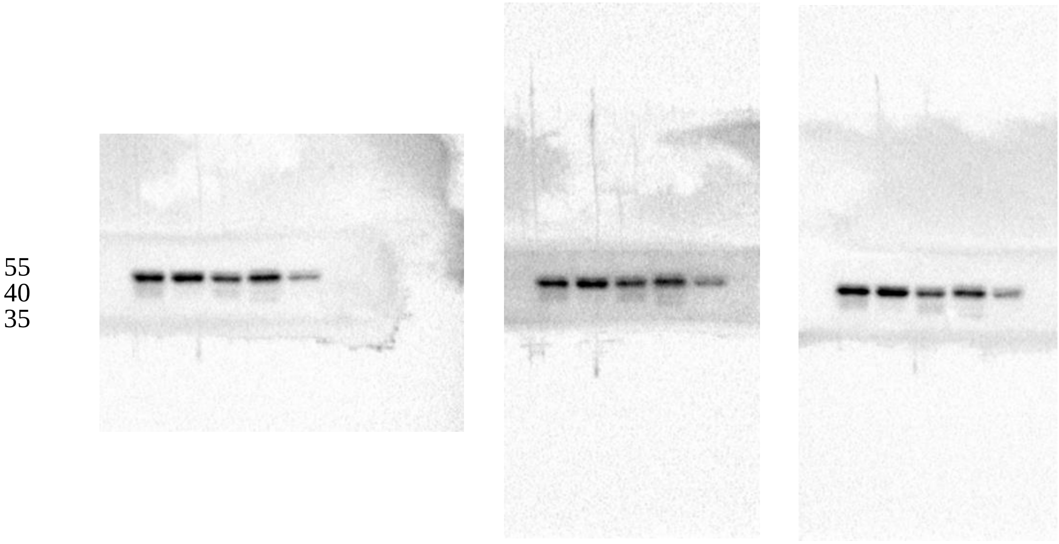

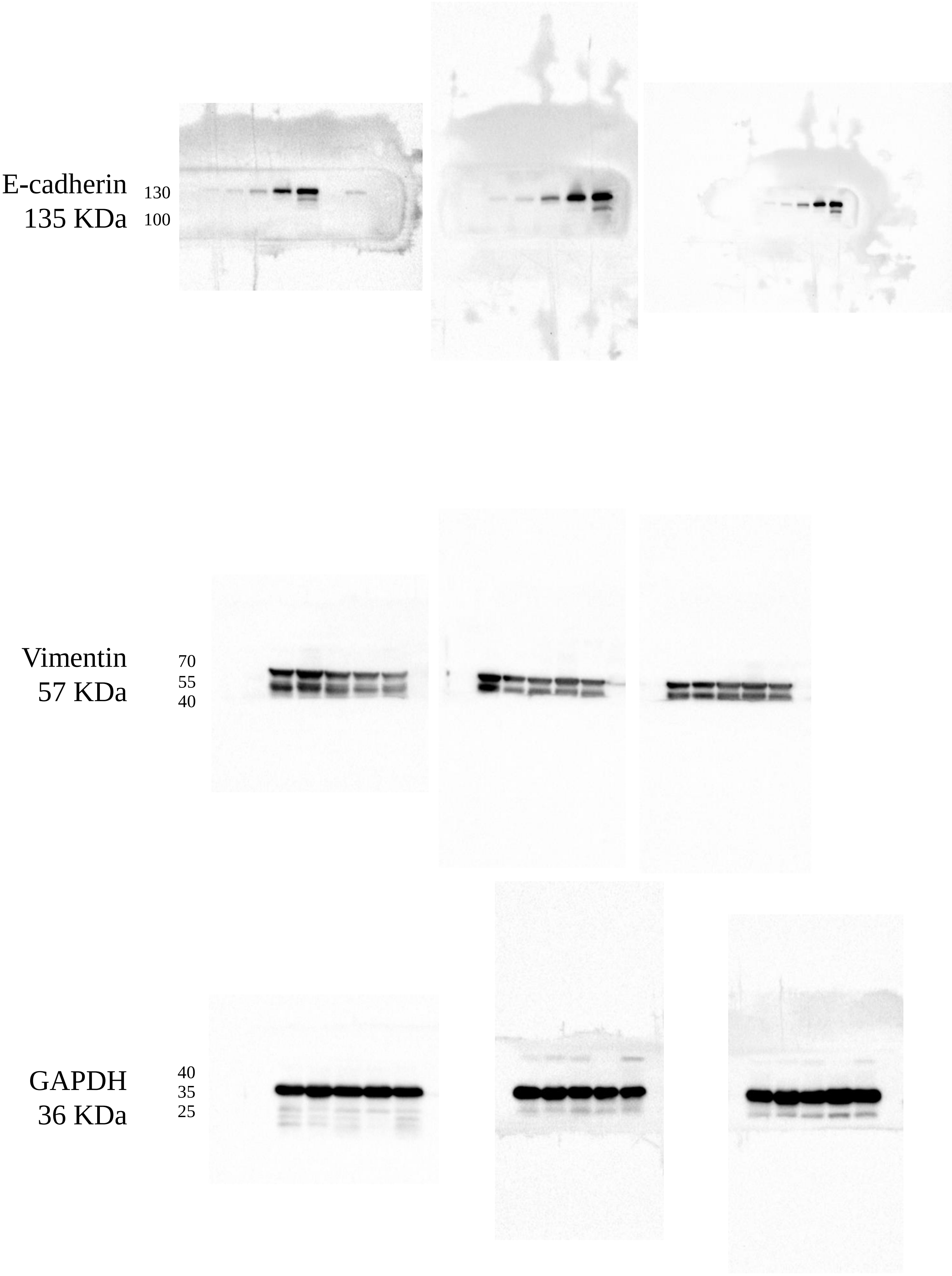

Supplementary Figure 1. The original, uncropped, and replicated blots were presented. The first column of blots correspond to cropped blots in the manuscript.
